# Septin 9 expression regulates ‘don't eat me’ signals and identifies an immune–epithelial class of intrahepatic cholangiocarcinoma

**DOI:** 10.1002/1878-0261.13673

**Published:** 2024-07-31

**Authors:** Ting ting Cai, Christophe Desterke, Juan Peng, Jean Agnetti, Peixuan Song, Dalila Ouazib, Alexandre Dos Santos, Catherine Guettier, Didier Samuel, Ama Gassama‐Diagne

**Affiliations:** ^1^ INSERM, Unité 1193 Villejuif France; ^2^ Université Paris‐Sud, Université Paris‐Saclay, UMR‐S 1193 Université Paris‐Sud, Université Paris Saclay, UMR‐S 1193 Villejuif France; ^3^ Université Paris‐Saclay, UFR Médecine‐INSERM UMR1310 Villejuif France; ^4^ AP‐HP Hôpital Paul Brousse, Centre Hépato‐Biliaire AP‐HP Hôpital Paul‐Brousse, Centre Hépato‐Biliaire Villejuif France

**Keywords:** ‘don't eat me’ signal, epithelial–mesenchymal transition, intrahepatic cholangiocarcinoma, septin 9, vimentin

## Abstract

Intrahepatic cholangiocarcinoma (iCCA) is a highly heterogeneous and aggressive liver cancer with limited therapeutic options. Precise classification and immunotherapy are perspectives to improve the treatments. We reported the role of septin 9 in apico‐basal polarity and epithelial‐to‐mesenchymal transition (EMT). Here, we aim to elucidate its role in iCCA. We analyzed single‐cell transcriptomes from human iCCA tumor cells based on phenotype and cell state. Knockdown of the septin 9 gene (*SEPT9*) was done using small interfering RNA (siRNA); interferon‐γ (IFN‐γ) stimulation was performed using different CCA cells; gene expressions were analyzed by reverse transcription and real‐time PCR analysis (RT‐qPCR); and immunofluorescence, immunoblotting, and flow cytometry were performed to assess the expression of proteins. The differential distributions of *SEPT9* and vimentin (*VIM*) gene expressions allowed us to define specific cellular trajectories of malignant cells and thus identified distinct clusters of iCCA cells. One cluster was enriched in *VIM* and extracellular‐matrix (ECM) remodeling molecules, and another had high expression of *SEPT9* and genes from the ‘don't eat me’ signal involved in immune escape. This antagonism between *SEPT9* and *VIM* was confirmed by *in vitro* experiments. Notably, *SEPT9* and ‘don't eat me’ gene expressions were inversely correlated to those of vimentin and the EMT markers. *SEPT9* expression was upregulated by IFN‐γ and *SEPT9* knockdown decreased expression of ‘don't eat me’ signal genes and increased expression of mesenchymal markers. Cancer Cell Line Encyclopedia (CCLE) transcriptome database analyses confirmed that iCCA cells enriched in septin 9 exhibit epithelial‐like features. This study revealed septin 9 as a cytoskeleton element of iCCA epithelial‐like cells and a regulator of the immune system response. It also brings new insights into the enigmatic relationship between EMT and immune response. Notably, we decoded a potential mechanism that could sensitize patients to immunotherapies.

## Graphical abstract

Septin 9 is identified as a regulator of EMT and the host immune response. Intrahepatic cholangiocarcinoma (iCCA) was categorized into two groups: (1) ‘immune–epithelial’, with high expression of septin 9 and ‘don't eat me’ signal genes regulated by IFNγ/STAT1; and (2) ‘mesenchymal–ECM’, with high expression of vimentin, MMP7 and TIMP1. Only the first group blocks immune cell attack.

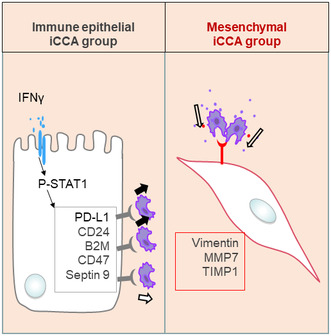

**Abbreviations**
Anti‐GD2: anti‐disialoganglioside
B2M: β‐2 macroglobulin subunit of the major histocompatibility class I complex
CAFs: cancer‐associated fibroblasts
CCA: cholangiocarcinoma
CCLE: Cancer Cell Line Encyclopedia
*CDH1*
: E‐cadherin gene
CK19: cytokeratin 19
CTRL: control
dCCA: distal cholangiocarcinoma
eCCA: extrahepatic cholangiocarcinoma
ECM: extracellular‐matrix
EMT: epithelial‐to‐mesenchymal transition
EpCAM: epithelial cell adhesion molecule
GEO: Gene Expression Omnibus
GO: Gene Ontology
GTP: guanosine triphosphate
HCC: hepatocellular carcinoma
HPC‐like: hepatic progenitor‐like cells
iCCA: intrahepatic cholangiocarcinoma
IFN‐γ/α/β: interferon‐γ/α/β
INF‐γ: interferon‐γ
MHC: major histocompatibility
MLL: mixed/lineage leukemia or mixed lineage leukemia
MT6‐MMP: membrane type 6 matrix metalloproteinase
NK: natural killer
NT: non‐tumor
*O*‐GlcNAc: *O*‐linked β‐*N*‐acetylglucosamine
PB: polybasic domain
PB2: second polybasic domain
PCA: principal component analysis
pCCA: perihilar cholangiocarcinoma
PD‐L1: programmed cell death ligand 1
pSTAT1: phosphorylation of STAT1
RPS6: ribosomal protein S6
RT‐qPCR: Reverse transcription and real‐time PCR analysis
S100A9: S100 calcium‐binding protein A9
SD: standard deviations
*SEPT9*
: septin 9 gene
siSeptin9: siRNA target *SEPT9*
TAM: tumor‐associated macrophages
TAMs: tumor‐associated macrophages
TECs: tumor‐associated endothelial cells
TIMP1: tissue inhibitor of metalloproteinases
TME: tumor microenvironment
t‐SNE: t‐distributed Stochastic Neighbor Embedding
*VIM*
: vimentin gene
*ZEB‐1*
: Zinc finger E‐box‐binding homeobox 1

## 1 Introduction

Cholangiocarcinoma (CCA) is a highly heterogeneous biliary aggressive carcinoma and the second most common primary liver cancer after hepatocellular carcinoma (HCC) [1]. According to the anatomical location, CCA is categorized as intrahepatic cholangiocarcinoma (iCCA), perihilar cholangiocarcinoma (pCCA) or Klatskin, and distal cholangiocarcinoma (dCCA). The incidence and mortality rate of iCCA increase worldwide, while the risk factors are less recognized than those for HCC. Increasing reports described shared risk factors between iCCA and HCC, including virus infections, alcohol, and metabolic syndrome [2]. Therapeutic options for patients with iCCA are limited, and liver resection and transplantation remain the only practical options. Unfortunately, only a few patients are eligible for surgery at the time of diagnosis. Treatments, including chemotherapy, give encouraging results in small cases but lack supports from large prospective trials [3]. Immunotherapy has opened a new area in cancer treatment, while its efficacy and safety remain unclear in iCCA. Thus, early and precise diagnosis and novel potential molecules for target treatment for iCCA is necessary.

The iCCA was considered as derived from cholangiocytes, the polarized epithelial cells lining the bile duct in the liver, while HCC was derived from hepatocytes. However, emerging literature revealed a more complicated scenario. Indeed, hepatocytes display remarkable plasticity and could be dedifferentiated and acquire stem cell traits contributing to both HCC and iCCA. Moreover, the cholangiocytes, in turn are plastic cells, could be dedifferentiated into stem‐like cells, increasing the aggressiveness and resistance to cancer treatment [4]. The epithelial‐to‐mesenchymal transition (EMT) is an essential driver of plasticity in cancer cells. EMT is a process through which epithelial cells lose their polarity and differentiation status to acquire mesenchymal, invasive features [5]. EMT and stem cell markers correlate to poor outcomes and are associated with lymph node metastasis in iCCA [6]. Thus, deciphering the molecular mechanisms which control plasticity‐EMT in iCCA cells is crucial for understanding its pathogenesis and identifying new therapeutic targets.

A typical hallmark of CCA is that cancer cells are embedded into a dense stroma containing fibrogenic cells, lymphatics and a variety of immune cells, of which cancer‐associated fibroblasts (CAFs) and tumor‐associated macrophages (TAM). Although the functional role of this rich stroma was not fully elucidated, studies suggest that the tumor microenvironment (TME) plays a key role in the progression and invasiveness of CCA [7]. Macrophages mediated‐cancer cell phagocytosis is often facilitated by intrinsic ‘eat me’ signals that function as ligands for phagocytic receptors, which can trigger intracellular signaling cascades, extensive remodeling of the cytoskeleton, and engulf the target cells. There are many types of ‘eat me’ signals such as phosphatidylserine (PS), mucins, and glycosylation and most of them are located on the cell surface, but some may be released outside the cell. These extracellularly released signals then bind to target cells [8].

Vimentin is a cytoskeletal filament expressed in mesenchymal cells [9, 10] and widely used as a marker of EMT. Vimentin is primarily located in the cytoplasm of cells. However, under circumstances like injury, stress, and senescence, vimentin can be expressed on the extracellular cell surface, or it can be released into the extracellular space. It was reported that vimentin functions as an engulfment receptor on neighboring phagocytes, which recognize *O*‐linked β‐*N*‐acetylglucosamine (*O*‐GlcNAc)‐modified proteins from apoptotic cells as ‘eat me’ ligands [11]. The polarized secretion of vimentin from the back of macrophage‐like cells, which strongly enhanced macrophage activation was recently reported [12]. Several studies confirmed an aberrant staining of vimentin in iCCA tumor cells while quite undetectable in benign cholangiocytes [13, 14, 15, 16, 17].

It has been discovered that proteins on the cell surface of cancer cells can tell macrophages not to eat and destroy them, thus using these ‘don't eat me’ signals to evade the immune system. To date, four members have been confirmed on the tumor surface, including CD47, programmed cell death ligand 1 (PD‐L1), the β‐2 macroglobulin subunit of the major histocompatibility class I complex (B2M), and CD24 [18, 19, 20, 21]. Except B2M, the three other proteins have been explored on iCCA cells [22, 23, 24]. Furthermore, monoclonal antibodies have been shown to have therapeutic potential for some cancers by antagonizing the interaction between ‘don't eat me’ signals and receptors expressed by macrophages [25, 26]. Anti‐CD47 has been shown to promote phagocytosis of macrophages, thereby inhibiting the growth and metastasis of iCCA. Besides, the unique overexpression of CD47 in iCCA but not HCC provides an opportunity for targeted immunotherapy [23]. Recent report revealed that the anti‐ disialoganglioside (Anti‐GD2) synergizes with CD47 blockade to mediate tumor eradication [27].

Using laser microdissection and proteomic to look for biomarkers for iCCA, our research team identified septin 9, a cytoskeleton element among the proteins with high expression in iCCA cells and undetectable in non‐tumor cells [13]. Septin 9 was first identified as a Myeloid Lymphoid Leukemia (MLL) fusion protein partner in acute myeloid leukemia [28] and was further implicated in different solid cancers [29, 30]. The quantification of methylated DNA at the promoter region of *SEPT9* in blood samples has been first proposed for early diagnosis of colorectal cancer [31]. Recently a panel of four DNA methylation markers, including *SEPT9* and *VIM*, has been reported in the diagnosis of CCA [32].

Septins form an evolutionally conserved family of guanosine triphosphate (GTP)‐binding proteins with 13 identified members [33]. Septins bind to the membrane through their polybasic domain (PB) and organize microtubules and actin cytoskeleton [34, 35], therefore, contributing to various biological processes, including cytokinesis, ciliogenesis, cell migration, vesicle trafficking, and cell polarity [36]. We identified a second polybasic domain in septins [35] and we demonstrated its role on organelle morphogenesis [34, 35, 37]. Furthermore, we reported that septin 9 expression and assembly through its two PB domains are essential for establishment and maintain of apico‐basal polarity against TGFβ‐dependent EMT [38].

In this study, using single‐cell RNA‐sequencing data from patients, we assess the expressions of septin 9 and vimentin, a classical EMT marker to seek for their potential contributions to iCCA heterogeneity. These studies were completed by *in vitro* experiments performed using CCA cell lines.

## 2 Materials and methods

### 2.1 Public datasets

Liver tumor single‐cell dataset GSE125449 was downloaded from the Gene Expression Omnibus (GEO) data access on the NCBI website. These data were processed with 10X Genomics technology in two sets of experiments performed on 19 liver tumor samples [39]. These experiments comprising a total of 9946 single‐cell transcriptomes were performed on nine tumor samples of hepatocellular carcinoma and 10 of iCCA.

### 2.2 Single cell analyses

Bioinformatics analyses were performed in r software environment version 4.0.2. single‐cell transcriptome preprocessing and cell heterogeneity was studied with seurat r‐package version 3.2.2 [40]. In the preprocessing step, genes with positive expression in a minimum of three cells were removed from the analyses in each set of experiments. The two sets of experiments were merged with canonical correlation batch correction. A Seurat object comprising 9946 transcriptomes with 21 287 features was created. Data were normalized and scaled before performing dimension reduction with principal component analysis (PCA) in 30 dimensions and with a t‐distributed Stochastic Neighbor Embedding (t‐SNE) algorithm in 15 dimensions of the previous PCA. Seurat object data were visualized with Dimplot, Ridgeplot, and FeaturePlot functions. Cell trajectory on cell compartment positive for *CK19* expression and from iCCA origin was performed with monocle package version 2.16.0 based on the alternative expressions of *SEPT9* and *VIM* [41]. Based on this cell distribution, a pseudotime transformation was performed to build a trajectory with the DDR Tree algorithm in the monocle 2 package. Branching analysis on intersection 2 of the tree was performed to characterize the gene cluster related to *SEPT9* expression and the gene cluster related to *VIM* expression in this trajectory. Functional enrichment performed on the Gene Ontology (GO) database was performed with ToppGene online application https://toppgene.cchmc.org [42].

### 2.3 Interactive online application

Additional resources with an interactive Web interface of the single cell analysis were built to facilitate data exploration of disturbing markers found on *SEPT9‐VIM* trajectory of tumor cells from iCCA. With markers that followed this cell trajectory, an interactive web interface was developed and is available at the following address: https://hsce.shinyapps.io/icca/. This interactive website was built with flexdasboard and shiny application inclusion. The user needs to select a gene identifier on the left sidebar, and the application will display the expression heterogeneity of this selected marker on a t‐SNE graph. Some positive cells for this marker will be displayed in the value box at the top right of the dashboard; also, barplot with some positive cells by groups, expression by the group will be displayed in violinplot, and finally, statistical summary (mean and standard deviation, SD) will be displayed by a group of samples.

### 2.4 Cell lines and culture conditions

HuCC‐T1 cells (RRID:CVCL_0324), derived from intrahepatic biliary tract, were kindly provided by Dr G. Gores (Mayo Clinic, MN). EGI‐1 cells (RRID:CVCL_1193), derived from extrahepatic biliary tract, were obtained from the German Collection of Microorganisms and Cell Cultures (DSMZ, Braunschweig‐Süd, Germany). Mz‐ChA‐1 cells (RRID:CVCL_6932), derived from extrahepatic biliary tract and the metastatic cell line SK‐ChA‐1 (RRID:CVCL_6952) were obtained from Dr A. Knuth (Zurich University, Switzerland). CC‐LP‐1 cells (RRID:CVCL_0205) derived from intrahepatic biliary tract were kindly provided by Dr Cedric Coulouarn (NuMeCan, Rennes, France). These different cells were cultured in DMEM supplemented with 1 g·L^−1^ glucose, 10 mmol·L^−1^ HEPES. HuH‐28 (RRID:CVCL_2955) cells derived from intrahepatic biliary tract, also kindly provided by Dr Cedric Coulouarn (NuMeCan), were cultured in MEM. All culture media were supplemented with Glutamax, 10% fetal bovine serum, antibiotics (100 UI·mL^−1^ penicillin and 100 mg·mL^−1^ streptomycin), and antimycotic (0.25 mg·mL^−1^ amphotericin B; Invitrogen, Gosselies,Belgium). All cell lines were routinely screened for the presence of mycoplasma by MycoAlert plus kit (#LT07‐170 Lonza, Basel, Switzerland).

The cells have been authenticated in the past 3 years by analyzing the main epithelial markers such as CK119, E‐cadherin. We also evaluated the mesenchymal and stem cells markers by RT‐qPCR to evaluate the potential changes. Importantly, the cells were implanted in nude mice and pathologist confirmed the CCA phenotype for EGI1, HuCCT1, and CC‐LP1.

### 2.5 siRNA and transfection reagents

siRNA for septin 9 were a Stealth RNAi™ siRNA (si1) (Cat#SEPT9HSS173896, Cat# SEPT9HSS173897) from Invitrogen, while non‐targeting siRNA (Cat#sc‐37007) came from Santa Cruz, LE VESINET, Ile De France. The sequences are presented below:

si Septin 9 (SEPT9HSS173896) (si2): 5′‐AGGCGCCUGCAUCACGGAACGAGAA‐3′, 5′‐UUCUCGUUCCGUGAUGCAGGCGCCU‐3′.

si Septin 9 (SEPT9HSS173897) (si3): 5′‐GCCAUGAAGCAGGGCUUCGAGUUCA‐3′, 5′‐UGAACUCGAAGCCCUGCUUCAUGGC‐3′.

The transfection of cDNA and siRNA was performed using jetPRIMETM (Ozyme), following the manufacturer's protocol.

### 2.6 Reverse transcription and real‐time PCR analysis (RT‐qPCR)

RNAs are extracted from cultured cells and tissue according to the protocol described by the manufacturers using the RNeasy Mini Kit 50 (Cat# 74104 QIAGEN,Marseille, France). The reverse transcription is then performed on 2 mg of RNA using the RevertAid™ First Strand cDNA Synthesis Kit (Cat#K1622 Fermentas, Burlington, Canada). The qPCR was then performed with the QuantiTect SYBR Green PCR Kit (Cat#204143 QIAGEN) and a Light Cycler 480 Real‐Time PCR System (Roche, Meylan, France). The cDNA was placed 10 min at min at 95 °C, followed by 45 cycles of 15 s at 95 °C, 30 s at 55 °C and 15 s at 72 °C. The glyceraldehyde‐3‐phosphate dehydrogenase (GAPDH) has been used as reference gene for normalization. Data represent mean for triplicate values for at least two independent experiments. The list of used primers is shown in Table S1.

### 2.7 Immunoblotting

The cells were washed twice using PBS and collected from the dish with 2× Laemmli sample buffer (4% SDS, 20% glycerol, 10% 2‐mercaptoethanol, 0.004% bromophenol blue, and 0.25 m Tris HCl, pH 6.8.) and placed at 96 °C for 10 min before loading on 10% SDS polyacrylamide gel and migrated at 40 mA per gel for about 60 min until the dye reaches the bottom the gel. After separation, the proteins were electro‐transferred onto nitrocellulose membrane at 100 V constant current. The membrane was stained with ponceau red to validate protein transfer. The membrane was rinsed with TBS containing 20 mm Tris, 150 mm NaCl 0.1% Tween 20 for 1 h at room temperature. Next, the membrane was incubated in TBS supplemented with 5% milk (TBSTM) to block non‐specific binding then with primary antibody (diluted in the TBSTM) overnight at 4 °C or for 2 h at room temperature, depending on the antibody. The membrane was washed three times for 5 min each with DPBS and incubated for 1 h at room temperature with the appropriate secondary antibody coupled with peroxidase. For detection, the ECL plus kit (Cat#32132 Thermo Scientific, Courtaboeuf Cedex, France) was used and the chemiluminescent signal was detected using the G:BOX Chemi Fluorescent & Chemiluminescent Imaging System from Syngene. Signal intensity was quantified using imagej software version 1.54 g.

### 2.8 Flow cytometry

Cells were detached from the plate using 100 μL of PBS buffer containing 2 mm EDTA (ethylenediaminetetraacetic acid). Cells were recovered in centrifuge tubes with PBS containing 5% fetal bovine serum (FBS). Cells were rinsed by two successive 5 min centrifugations at 900 ***g*** g with PBS containing FBS. Washed cells were resuspended with the primary antibody for 1 h at 37 °C and, after washing, incubated with the secondary antibody for 1 h at 37 °C. Fluorescence intensity was measured by flow cytometry using bd accuri c6 plus software, San Jose, CA, USA, and data were determined using live cells and subtracting background (isotype).

### 2.9 Immunofluorescence

This was performed as previously reported [34]. The cells were cultured on coverslips, fixed for 20 min at 4 °C with 4% paraformaldehyde, and permeabilized for 30 min at 37 °C with the permeabilizing buffer: PBS with 0.025% and 0.7% m.v‐1 gelatin from cold water fish skin (PFS) (Cat#G7041 Sigma, Saint‐Quentin‐Fallavier Cedex, France), as well as saponin (Cat#10294440 Fisher Scientific). After that, the cells were treated for 2 h with primary antibodies. They were then incubated with the appropriate secondary antibodies or with the dye for 90 min after being cleaned three times for 5 min each time using PFS. We used Prolong Gold (Cat#P36934 Invitrogen) to mount the coverslips. A Leica TCS SP5 AOBS tandem confocal microscope was used to capture the images.

### 2.10 Antibodies

Rabbit anti‐septin 9 Cat#ab38314 (WB: 1/500, IF: 1/25), mouse and Anti‐B2M antibody Cat#ab75853 (WB: 1/5000, IF: 1/80) were obtained from Abcam, Paris, France; goat anti‐PD‐L1 Cat#AF156 (WB: 1/1000, IF: 1/50) came from R&D Systems, Minnneapolis, MN, USA; goat anti‐Actin Cat#sc‐1616 (WB: 1/1000) was obtained from Santa Cruz Biotechnology. Mouse anti‐Vimentin CBL202 were obtained from Millipore, Burlington, USA (IF: 1/100). Anti‐mouse IgG‐HRP and anti‐rabbit IgG‐HRP came from GE Healthcare, Solingen, Germany (WB: 1/1000). Anti‐goat IgG‐HRP Cat#sc‐2020 (WB: 1/1000) was obtained from Santa Cruz Biotechnology; Alexa Fluor 568 Cat#A11004, A11011 and A11057 (IF: 1/100), Alexa Fluor 488 Cat#A11001, A21206 and A21202 (IF: 1/100) are from Invitrogen, Actin was stained with Alexa Fluor 594 phalloidin Cat#A12381 (IF: 1/100) and nuclei with Hoechst Cat#H21486 (IF: 1/10 000), both from Invitrogen. Rabbit anti‐pSTAT1 (Tyr701) was from Cell Signaling, Saint‐Cyr‐L'École, France.

### 2.11 Immunohistochemical analysis

Sections from a tissue microarray (TMA) described in a precedent report [13] were used for immunohistochemical examination. After the proper antigen retrieval procedure, the deparaffinized 4‐μm‐thick sections were incubated with primary antibodies against septin 9, vimentin, and PD‐L1 for a full night at 4 °C. With Vector Laboratories' Vectastain ABC technique, primary antibody/antigen interaction was identified. Hematoxylin was used as a stain for the nuclei. By not using the primary antibody during the experiment, labeling specificity was ascertained. Using the axio vision rel. 4.5 software, Los Angeles, CA, USA, images were captured with the CCD camera Axio‐Imager.M1 (Zeiss, Oberkochen, Germany). The percentage of positive tumor cells on each slide (0–100%) was multiplied by the dominant staining intensity (0 = negative, 1 = weak, 2 = intermediate, and 3 = strong) to obtain the labeling scores. Scores that were obtained varied from 0 to 300. Negative, weak (+), moderate (++), and strong‐level expression (+++) were the classifications assigned to specimens with total scores ranging from 0 to 5, 6 to 50, 50 to 119, and 120 to 300, respectively. Among these, group Low comprises tissue samples with negative and weak expression of the stained protein, whereas group High comprises tissue samples with moderate and high expression of the stained protein. This enabled the automation of deparaffinization, immunohistochemistry, *in situ* hybridization, and antigen retrieval with a polymer‐peroxidase‐DAB detection kit. Triplicate tissue sections were prepared for each stain. Sections of tissue were immunostained with primary antibodies against CD3 (Dako Monoclonal, Santa Clara, CA, USA) or CD8 (Dako Monoclonal) on an Autostainer BOND MAX (Menarini, Firenze, Italy) to analyze CD3 and CD8. This enabled the automation of deparaffinization, immunohistochemistry, *in situ* hybridization, and antigen retrieval with a polymer‐peroxidase‐DAB detection kit. Pathologists rated each immunohistochemistry staining result.

The study methodologies conformed to the standards set by the Declaration of Helsinki.

The experiments were undertaken with the understanding and written consent of each subject with approval of the institutional review board of the INSERM (Institutional Review Board Number 11‐047).

### 2.12 Statistical analyses

Statistical significance of RT‐qPCR, immunofluorescence immunoblotting and flow cytometry were determined by Student's *t*‐test using Microsoft Office Excel software (Microsoft Corporation, Redmond, Washington , USA). The displayed results showed the means and the SD (standard deviations), and those with *P* values < 0.05 were considered statistically significant (*P < 0.05, **P < 0.01, ***P < 0.001, *t*‐test). All tests are two‐slide.

## 3 Results

### 3.1 *SEPT9* and *VIM* expressions are higher in iCCA cells than in HCC cells

**Fig. 1:**
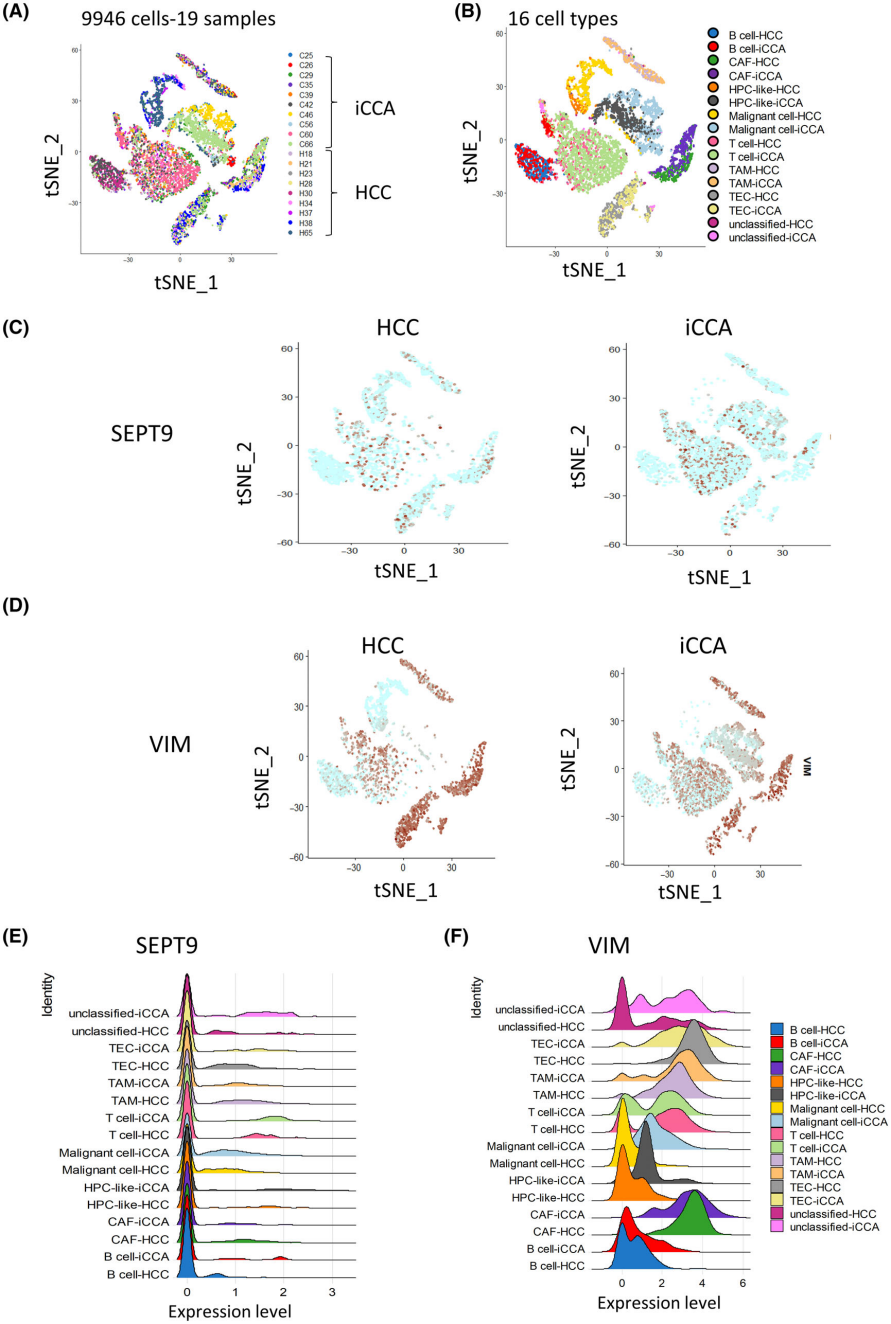
Higher expression of *SEPT9* in iCCA cells than in HCC cells. (A) t‐SNE dimension reduction of the 9946 liver cancer cells stratified by their phenotype characterization. (B) t‐SNE dimension reduction of the 16 liver cancer cells stratified by their phenotype characterization. (C, D) t‐SNE DimPlot of *SEPT9*/*VIM* expression in cells from liver tumors stratified by their HCC and iCCA origins. (E, F) Ridegeplot of the single‐cell transcriptome showing the expression of *SEPT9*/*VIM* in liver cancer samples. (HCC, hepatocellular carcinoma; iCCA, intrahepatic cholangiocarcinoma; *SEPT9*, septin 9 gene; t‐SNE, t‐distributed Stochastic Neighbor Embedding; *VIM*, vimentin gene).

First, we assessed the heterogeneity of iCCA cells by processing the single‐cell transcriptome of liver cells from biopsy tumor samples in the GSE125449 dataset comprising nine samples of HCC and 10 samples of iCCA [39]. The cells were annotated based on the best‐known lineage‐specific marker genes for each of the eight cell types, including CAFs, tumor‐associated endothelial cells (TECs), T cells, B cells, tumor‐associated macrophages (TAMs), cells with hepatic progenitor‐like cells (HPC‐like), cancer cells/malignant cells and unclassified cells (Fig. S1). Dimension reduction performed with the t‐SNE algorithm allowed us to stratify 9946 transcriptomes according to their tumor origins (HCC or iCCA) and their cell types (Fig. 1A,B). The expression of cytokeratin 19 (*CK19*) was used to identify iCCA (Fig. S2A), which was restricted to HPC‐like and malignant cells from iCCA tumor origins (Fig. S2A), in accordance with previous studies [43, 44].

Next, we investigated the expression of *SEPT9* in this single‐cell transcriptome (Fig. 1C,E). Septins cellular functions are often associated with their hetero‐oligomerization to form filaments [45]. Thus, we performed investigations of other *SEPTs* expressions in human iCCA tumor cells. Among the *SEPTs*, we highlighted *SEPT7* (Fig. S2C), and *SEPT2* (Fig. S2D), which are the more highly expressed septins and are present in the same filament structure as *SEPT9* [46]. These analyses revealed that *SEPT7* (Fig. S2C) and *SEPT2* (Fig. S2D) are expressed in different types of cells, such as T cells, tumor endothelial cells, malignant cells, and CAFs, as shown for *SEPT9* (Fig. 1C,E). Interestingly, the expression of *SEPT9* appeared to be higher in malignant cells of iCCA origin than in those of the same type from HCC origin (Fig. 1C,E). *SEPT9* was also exceptionally well expressed in T cells, in line with its first description in MLL [28]. However, *SEPT7* was higher in CAFs from HCC than an iCCA origin (Fig. S2C), and *SEPT2* had no marked difference in expression between cells from HCC and iCCA (Fig. S2D). These results indicated different cellular expressions of septins in liver cancer cells, with higher expression in cells from iCCA than HCC.

Finally, to assess EMT, we explored the prevalence of E‐cadherin, the prototypic epithelial marker, and vimentin as a mesenchymal marker. The E‐cadherin gene (*CDH1*) expression was very low and mainly expressed in malignant cells, especially in iCCA samples (Fig. S2B). In contrast, to *SEPT9*, *VIM* expression was very high in both HPC‐like cells and malignant cells as well as in T cells and stromal cells (Fig. 1C–F).

### 3.2 *SEPT9* and *VIM* are differential expressed in iCCA cells

**Fig. 2:**
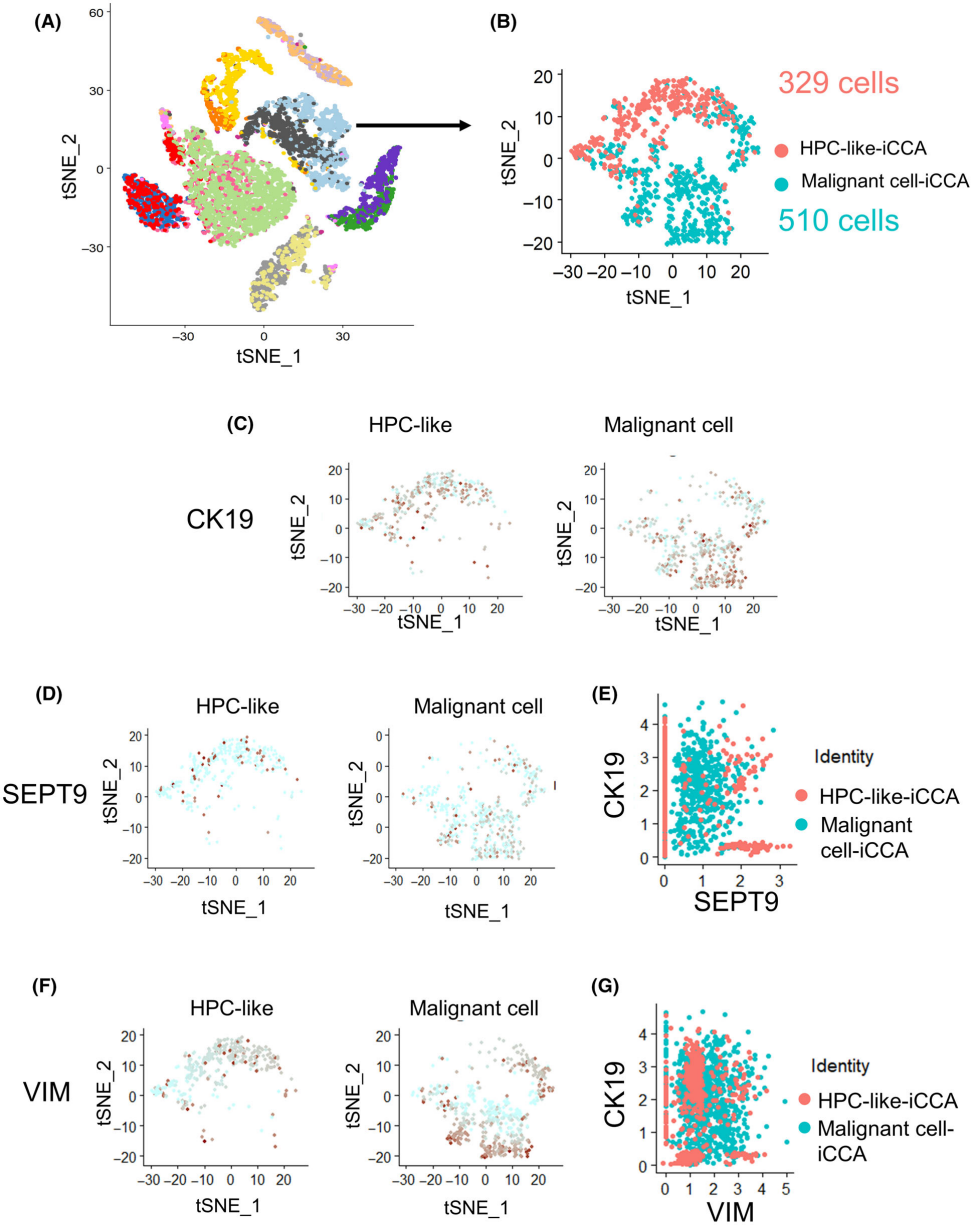
Analysis of *VIM* and *SEPT9* expression in *CK19*‐positive iCCA tumor cells. (A) Subset procedure to analyze tumor cells of iCCA origin in the single‐cell transcriptome in liver cancer samples. (B) t‐SNE DimPlot resulting in a subset of 839 iCCA tumor cells comprising the following cell phenotypes: malignant cells and HPC‐like. (C) t‐SNE DimPlot of the *CK19* expression subset of iCCA tumor cells stratified by their phenotype. (D, F) t‐SNE DimPlot of *SEPT9*/*VIM* expression in iCCA tumor cells stratified by their phenotype. (E, G) Scatterplot of *CK19* versus *SEPT9*/*VIM* expression in iCCA tumor cells. (CK19, cytokeratin 19; iCCA, Intrahepatic cholangiocarcinoma; *SEPT9*, septin 9 gene; t‐SNE, t‐distributed Stochastic Neighbor Embedding; *VIM*, vimentin gene).

To further investigate the diversity of iCCA malignant cells, a subset of the single‐cell transcriptome was built comprising HPC‐like cells and malignant cells expressed *CK19* (Fig. 2A,B) from iCCA. Therefore, we focused the study on 329 HPC‐like cells and 510 malignant cells from iCCA (Fig. 2B) based on t‐SNE analysis. First, the *CK19*‐positive cell distribution in these two types of cells was analyzed (Fig. 2C). To note, *CDH1* was expressed in the compartments of both HPC‐like and malignant cells (Fig. S3A). Scatterplot analysis showed important proportion of *CDH1* ^+^ *CK19* ^+^ double positive cell population in these types of tumors (Fig. S3B). Then, to examine *SEPT9* and *VIM* expression, we performed a t‐SNE analysis in these two types of cells. The expression of *SEPT9* was higher in HPC‐like cells than in malignant cells (Fig. 2D). Effectively, *SEPT9* high–*CK19* high cells concerned the HPC‐like cell compartment (Fig. 2E). In contrast, the malignant cells expressed high levels of *VIM* (Fig. 2F,G). The *SEPT9* high and the *VIM* high cell compartments implicated both HPC‐like and malignant cells populations (Fig. 2E,G). These results suggested that the differential expression of *SEPT9* and *VIM* may contribute to the heterogeneity in iCCA tumors.

### 3.3 Alternative expression of *SEPT9* and *VIM* defines a specific cell trajectory in iCCA tumor cells

**Fig. 3:**
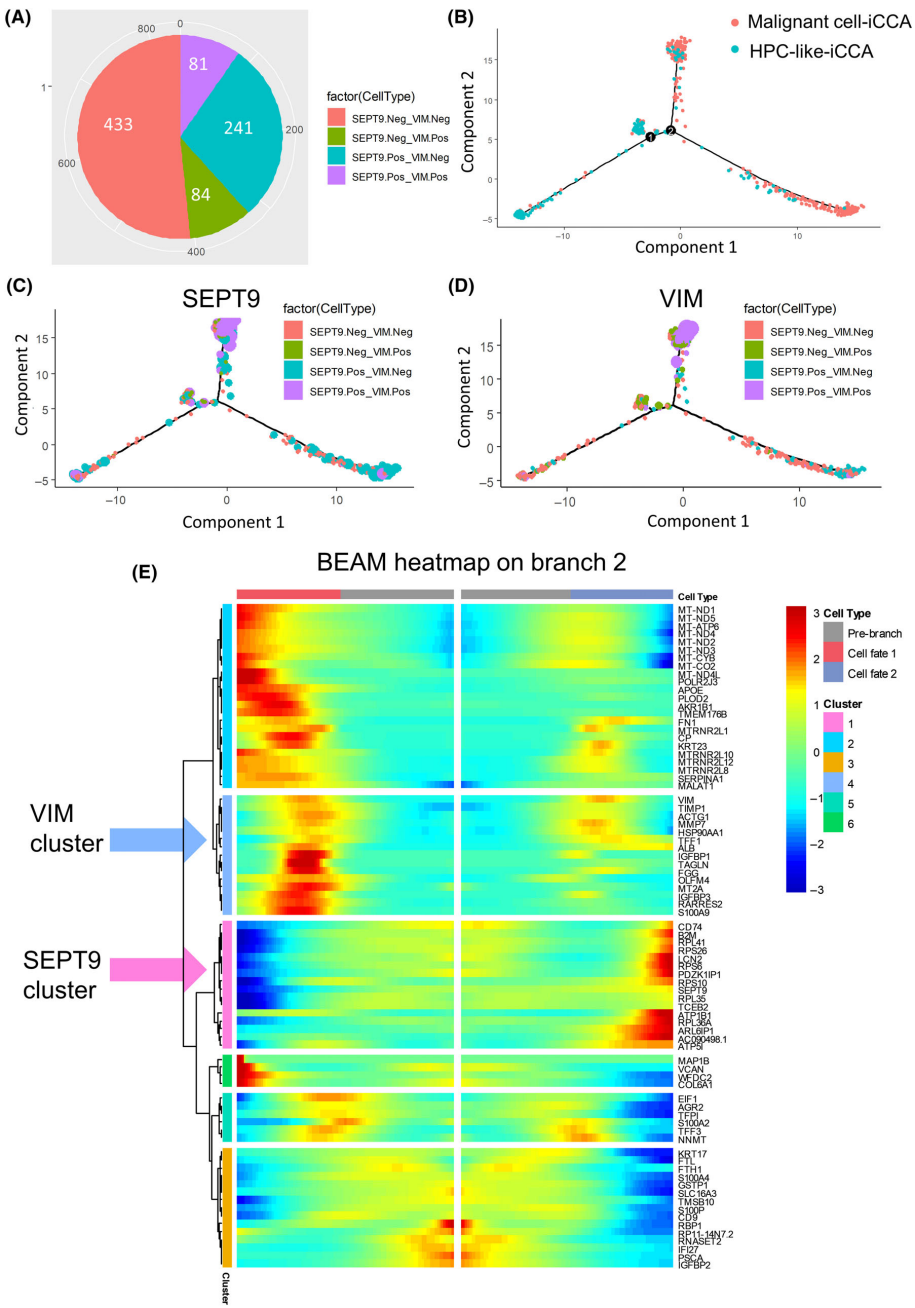
Alternative expression of *SEPT9* and *VIM* defines a specific cell trajectory in iCCA tumor cells. (A) Pie chart of the alternative expression of *VIM* and *SEPT9* in iCCA tumor cells. (B) Pseudotime tree transformation based on the alternative expression of *VIM* and *SEPT9* in iCCA tumor cells (distinct colors represent distinct cell types). (C/D) Pseudotime tree with *SEPT9*/*VIM* expression (point size) and *SEPT9‐VIM* group definition (4 distinct colors defined on the pie chart in A). (E) Pseudotime heatmap of the main markers found on the cell trajectory after branching analysis on intersection 2 of the tree. (iCCA, intrahepatic cholangiocarcinoma; *SEPT9*, septin 9 gene; *VIM*, vimentin gene).

In single‐cell RNA‐sequencing experiments, cells could represent progressively changing states along a biological process. A helpful approach to analyzing data from these experiments is to compute computationally ordering cells based on gene expression transition. The ordered cells represent samples along pseudotime trajectories [47]. Groups were defined in 839 iCCA tumor cells based on *SEPT9* and *VIM* expression divergence, yielding 433 double‐negative cells, 241 *SEPT9* ^+^/*VIM* ^−^ cells, 84 *SEPT9* ^−^/*VIM* ^+^ cells, and 81 double‐positive cells (Fig. 3A,B). Pseudotime transformation allowed us to reconstitute a tree cell trajectory (Fig. S4A), aiding in stratifying iCCA malignant cells from iCCA HPC‐like cells, especially on branching number 2 (Fig. 3A and Fig. S4B). Expression heterogeneity of *SEPT9* (Fig. 3C) and *VIM* (Fig. 3D) respected this pseudotime transformation and group definition. Branching analysis at intersection number 2 identified a specific trajectory profile (Table S1) with molecules following *VIM* expression (Fig. 3E) and those following *SEPT9* expression during the cell trajectory (Fig. 3E). This analysis highlighted the importance of this expression trajectory, especially for cell fate decisions between iCCA malignant cells and iCCA HPC‐like cells, as shown previously (Fig. 3A). The reconstitution of cell trajectory also revealed variation in *CK19* (Fig. S5A,B), epithelial cell adhesion molecule (*EPCAM*) an epithelial marker and *CDH1* (Fig. S5E,F) during the pseudotime transformation. The t‐SNE analysis confirmed important expression of *CDH1* in iCCA tumor cells both in compartments HPC‐like and malignant cells (Fig. S2B) and the scatterplot analysis highlighted a substantial *CDH1* ^+^ *CK19* ^+^ double‐positive cell population in iCCA (Fig. S3B).

The heatmap (Fig. 3E) from genes involved in branching number 2 in Fig. 3B, revealed that the *VIM* trajectory cluster mainly implicated molecules associated with signaling receptor binding: *a tissue inhibitor of metalloproteinases* (*TIMP1*), *S100 Calcium Binding Protein A9* (*S100A9*) (Fig. 3E). The *SEPT9* trajectory cluster mainly implicated molecules associated with structural components of the ribosome, such as *ribosomal protein S6* (*RPS6*) and surprisingly we also found molecules involved in immunity, such as *CD74* and the *β‐2 macroglobulin subunit of the major histocompatibility class I complex* (*B2M*) (Fig. 3E). To better interpret the involvement of these markers in the transition between *VIM* ^+^ and *SEPT9* ^+^ cells within iCCA tumor cells, an internet interface was developed at the following address: (https://hsce.shinyapps.io/icca/) (Fig. S6). This web interface through t‐SNE analysis confirmed that positive expression of *SEPT9* is larger than *VIM* expression in iCCA malignant cells. *CD74* expression was similar to that of *SEPT9*. Effectively, a subset of *VIM* ^+^ cells (Fig. S7A) was *CD74* negative (Fig. S7D) and presented high levels of invasive markers, such as *TIMP1* (Fig. S7C) and *MMP7* (Fig. S7E). These results suggested that the expression of *SEPT9* (Fig. S7B) and *VIM* (Fig. S7A) in iCCA tumor cells could define a cell trajectory that stratified HPC‐like cells from malignant cells. We highlighted a larger subset of *SEPT9* (*CD74* ^+^) cells with an immunological activity that implicated *B2M* in these tumor cells (Fig. S7F). According to GO database analysis, the functions of the *SEPT9* cluster and *SEPT9* cluster were further confirmed. We noted that *B2M*, *CD74*, ribosomal components, co‐translational protein targeting to the membrane were in the *SEPT9* cluster (Fig. S8A,C), while the *VIM* cluster was prominently associated with defense response to organism, the cell migration, regulation of exocytosis, and secretion (Fig. S8B,D). These new findings strongly suggested a role of *SEPT9* in the regulation of the immune system.

### 3.4 Expressions of ‘don't eat me’ signals genes are higher in iCCA than in HCC and are independent parameters to predict iCCA whatever the age and gender of the patients

**Fig. 4:**
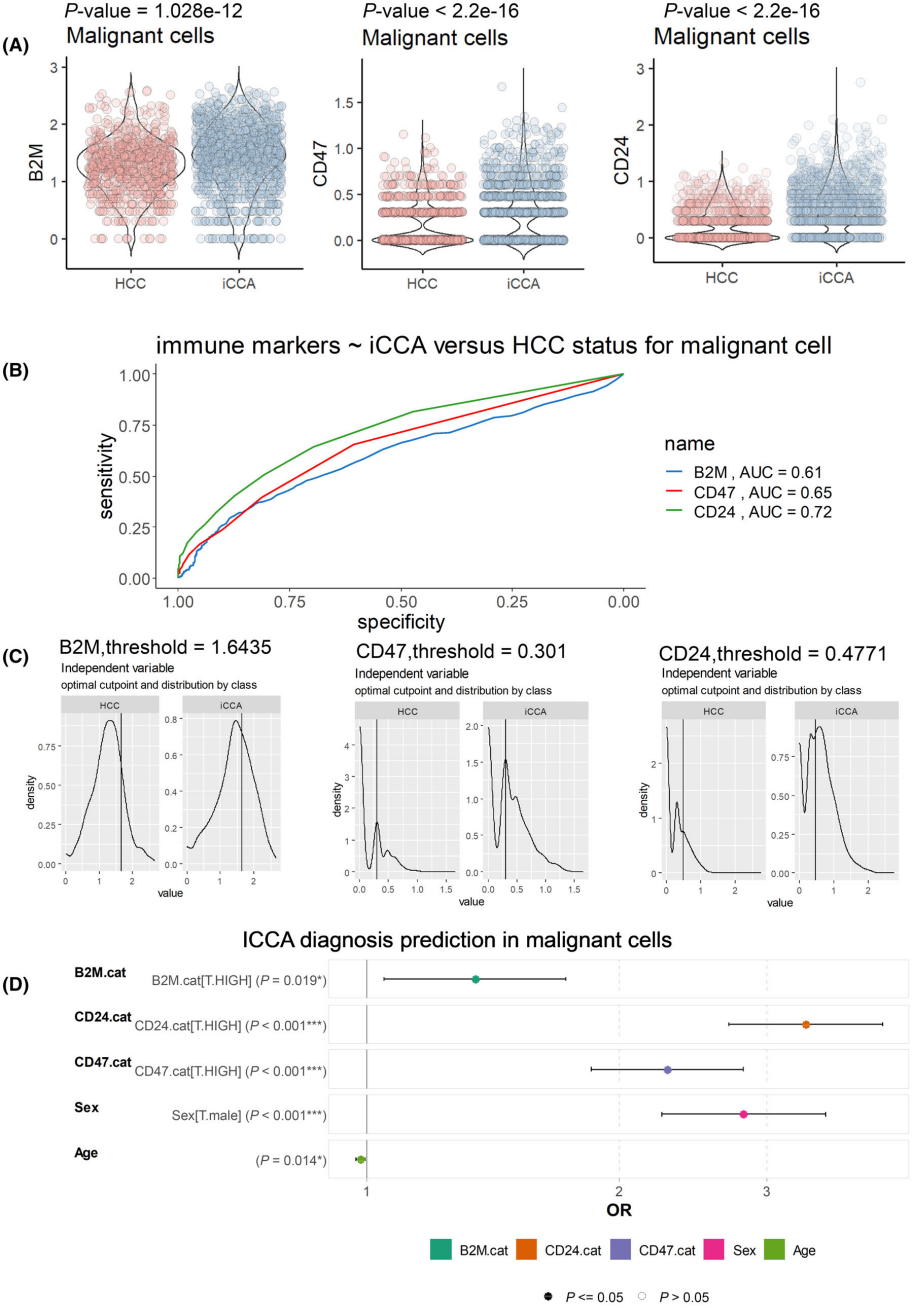
Single cell expression of ‘don't eat me’ signals (B2M, CD47, CD24) in malignant cells is an independent predictor of iCCA diagnosis among liver cancers. (A) Respective violin plots with unpaired two‐sided *t*‐test evaluating single cell expression of B2M, CD47 and CD24 in malignant cells according liver cancer diagnoses: HCC, hepatocellular carcinoma; iCCA, intrahepatic cholangiocarcinoma; (B) ROC curve analyses performed on single cell expression of B2M, CD47, and CD24 in malignant cells testing prediction of iCCA diagnosis as compared to HCC diagnosis (AUC, area under the curve); (C) Optimal threshold determination to discretize expression of B2M, CD47, and CD24 in malignant cells according to iCCA diagnosis (ROC, receiver operating characteristic); (D) Multivariate logistical model predicting iCCA diagnosis with inclusion of B2M, CD27, and CD47 single cell expression in malignant cells but also age and gender (sex) of respective patients (*P < 0.05, ***P < 0.001, statistical *P*‐values were obtained by multivariate GLM: generalized linear model with ‘logit’ family). B2M.cat: expression categories of β‐2 macroglobulin subunit of the major histocompatibility class I complex (‘T’: group control with low expression level versus ‘HIGH’ expression level); CD24.cat: expression categories of CD24 (‘T’:group control with low expression level versus ‘HIGH’ expression level); CD47.cat: expression categories of CD47 (‘T’:group control with low expression level versus ‘HIGH’ expression level); Sex: gender of patients (‘T’: group control (female) versus ‘male’ group); Age: age of the patients. Error bars represent confident interval at 95 percent (IC95) (AUC, area under the curve; HCC, hepatocellular carcinoma; iCCA, Intrahepatic cholangiocarcinoma; OR, odds ratio).

Comparative single cell expression analyses of ‘don't eat me’ molecules such as *B2M*, *CD47*, and *CD24* were performed in malignant cells according to liver cancer subgroup diagnoses: iCCA versus HCC. These analyses revealed a significant increase of the expressions of these three molecules in malignant cells from iCCA compared to those of HCC cells (Fig. 4A): *B2M* (*P*‐value = 1.028e‐12), *CD47* (*P*‐value < 2.2e‐16), and *CD24* (*P*‐value < 2.2e‐16). Their single cell expressions in malignant cells were also investigated in individual ROC (Receiver operating characteristic) curve analyses according to iCCA diagnosis compared to HCC ones (Fig. 4B). For each ‘don't eat me’ signal molecule, positive ROC curve for iCCA prediction was observed with a respective area under curve (AUC): *B2M* (AUC = 0.61), *CD47* (AUC = 0.65), and *CD24* (AUC = 0.72). In order to evaluate whether these three molecules are independent markers of iCCA diagnosis, their single cell expressions were discretized at an optimal threshold for iCCA diagnosis (Fig. 4C). A multivariate logistical model for iCCA diagnosis prediction was not only built on binomial family of generalized linear model with inclusion of *B2M*, *CD47*, and *CD24* single cell expression in malignant cells but also epidemiological parameters such as age and gender (sex) of patients (Fig. 4D). This multivariate predictive model highlighted that single cell expression of *B2M* (multivariate *P*‐value = 0.019), *CD47* (multivariate *P*‐value < 0.001), and *CD24* (multivariate *P*‐value < 0.001) are independent markers of prediction for iCCA diagnosis whatever the age and sex of the patients.

### 3.5 *SEPT9* is enriched in a wide range of iCCA cells with epithelial and immune features

To underpin the relationship between septin 9 and the immune system and its opposed expression with vimentin, we analyzed a group of 18 iCCA cell lines from the CCLE database. First, we performed PCA of *SEPT9* and *VIM* expression, and the cells were divided into three subgroups: *SEPT9* ^+^/*VIM* ^−^, *SEPT9* ^−^/*VIM* ^+^, and *SEPT9* ^+^/*VIM* ^+^ (Fig. S9A). The cell lines were listed in the subgroups, and their expression levels of *SEPT9* and *VIM* are presented as bar graphs (Fig. S9B). In the *SEPT9* ^+^/*VIM* ^−^ subgroup, we further observed enrichment in cellular components related to the cell polarity (Fig. S9C), which indicated septin 9 was highly related to epithelial features. The analysis for signaling evidenced immune biological processes, especially cytokine signaling in the immune system and interferon γ/α/β (IFN‐γ/α/β) signals (Fig. S9D). Furthermore, the expression of the genes related to the above signals between the *SEPT9* ^+^/*VIM* ^−^ and *SEPT9* ^−^/*VIM* ^+^ subgroups is presented in Fig. S9E, and we clearly observed higher expression of *B2M*, *CD74*, *IRF (1,2,5,6,8)*, and *STAT2* in the *SEPT9* ^+^/*VIM* ^−^ group than in the *SEPT9* ^−^/*VIM* ^+^ group. All these data highlighted *SEPT9* as a marker of epithelial and immune group of iCCA and suggested its role in the regulation of the ‘don't eat me’ signaling by IFN signal. These results also confirmed that *SEPT9* and *VIM* expression patterns could serve to classify iCCA.

### 3.6 Septin 9 and vimentin expression patterns characterized EMT and immune escape signals in CCA cells

Vimentin is a cytoskeletal protein at the center of the EMT‐mediated metastasis [48]. The data above revealed that septin 9 and vimentin could separate the iCCA into different cell clusters (Fig. 3E). We recently reported that septin 9 negatively regulated EMT in line with above data which revealed the enrichment of septin 9 and vimentin in different CCA (Fig. 3E). Accordingly, we investigated the relation between septin 9 expression and EMT in CCA cells. Hence, we used six CCA cell lines from different origins we disposed including iCCA cells such as HuCC‐T1, HuH‐28, CC‐LP‐1, extrahepatic biliary tract cells (eCCA), Mz‐ChA‐1and EGI‐1, and metastatic cell line, SK‐ChA‐1. Interestingly septin 9 is highly expressed in these eCCA cells.

**Fig. 5:**
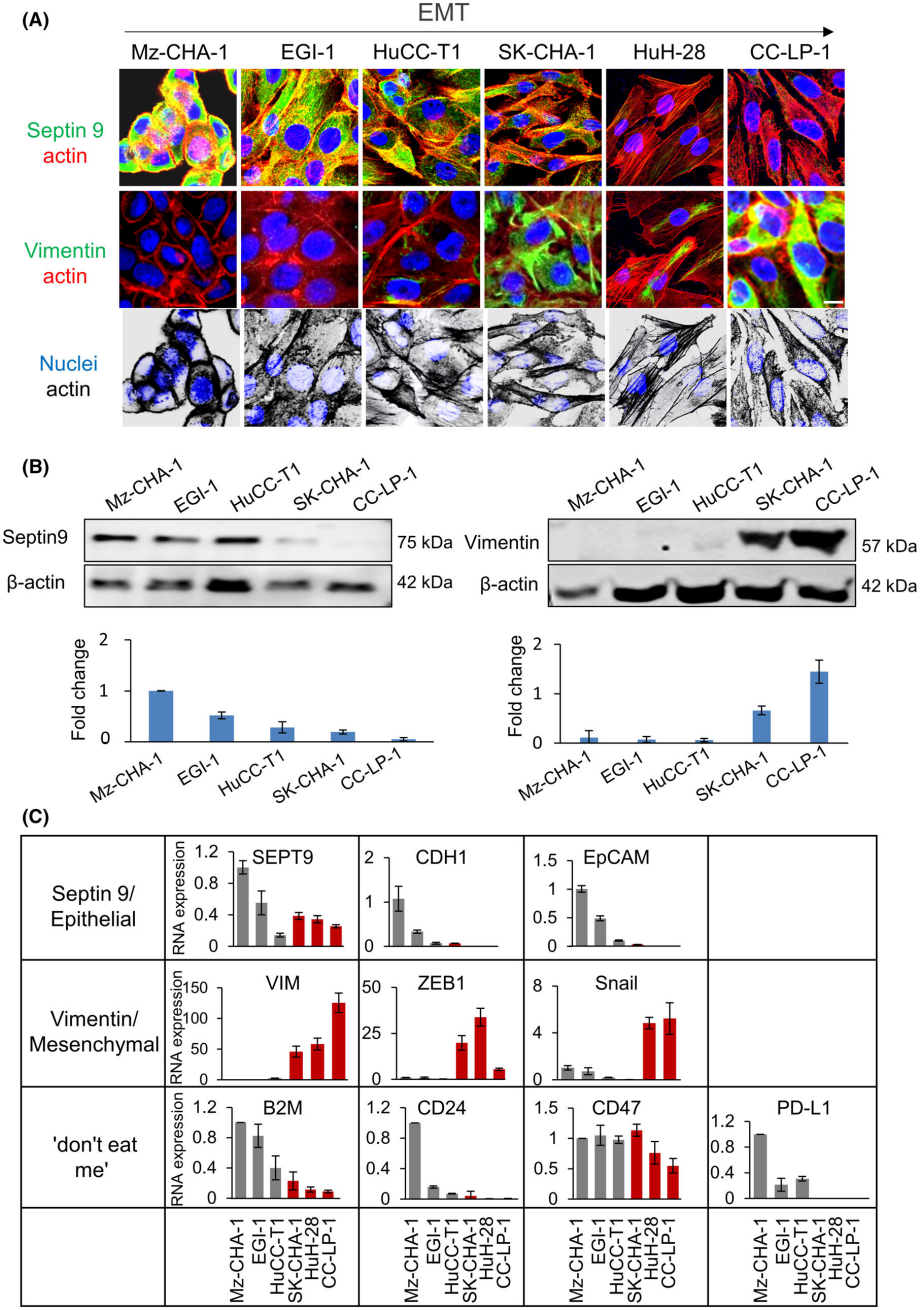
Septin 9 expression is correlated to EMT and ‘don't eat me’ signal in CCA cells. (A) The cells were grown for 24 h and then stained for septin 9 (green), actin (red), vimentin (green), and actin (red). Scale bar, 10 μm, *n* = 3. (B) The cell lysates from (A) except HuH‐28 were analyzed by immunoblot for septin 9 and vimentin. β‐actin was used as a loading control. The signal intensities were presented as bars below, *n* = 3. (C) qRT–PCR analysis for *SEPT9*, *CDH1*, *EpCAM*, *VIM*, *ZEB1*, *Snail*, and ‘don't eat me’ signaling mRNA expression of Mz‐ChA‐1, EGI‐1, HuCC‐T1, SK‐ChA‐1, HuH‐28, and CC‐LP‐1, *n* = 3. GAPDH was used as a loading control, and the fold change was compared to that of Mz‐ChA‐1. All data represented mean ± SEM (Student's *t*‐test). (CCA, cholangiocarcinoma; *CDH1*, E‐cadherin gene; EMT, epithelial‐to‐mesenchymal transition; *EpCAM*, epithelial cell adhesion molecule; RT‐qPCR, Reverse Transcription and Real‐time PCR analysis; *SEPT9*, septin 9 gene; *VIM*, vimentin gene; *ZEB‐1*, Zinc finger E‐box‐binding homeobox 1).

We performed immunofluorescence analysis which revealed that septin 9 expression was very strong in Mz‐ChA‐1 and EGI‐1, its decreased in the other cells and was very low in CC‐LP‐1 which presented a very high signal of vimentin (Fig. 5A). Actin‐stained revealed a round or polygonal shape for the epithelial cells (Mz‐ChA‐1, EGI‐1, and HuCC‐T1) with extensive cell–cell contacts. In contrast, mesenchymal cells (SK‐ChA‐1, HuH‐28, and CC‐LP‐1) presented spindle‐shaped and had important stress fibers (Fig. 5A). We confirmed the inverse expression of septin 9 and vimentin in CCA cells by performing immunoblots (Fig. 5B). Furthermore, the inverse expression profile of septin 9 and vimentin was verified at the mRNA level using RT‐qPCR (Fig. 5C). Then, we analyzed other epithelial markers including EpCAM and *CDH1* and mesenchymal markers including Zinc finger E‐box‐binding homeobox 1 (*ZEB1*) and *SNAIL* (Fig. 5C). The expression of these different markers allowed us to define one group of CCA cells with high expression of epithelial markers which include Mz‐ChA‐1, EGI‐1, HuCC‐T1, SK‐ChA‐1 and the other group with mesenchymal features such as HuH‐28 and CC‐LP‐1 which were enriched in vimentin and *ZEB‐1*, indicating that septin 9 was enriched in cells with epithelial features and decreased in cells with mesenchymal features that expressed vimentin. Lately, the ‘don't eat me’ genes were also analyzed by the RT‐qPCR (Fig. 5C). As expected, these genes were highly expressed in the septin 9‐enriched cells while their expression decreased in the vimentin‐enriched mesenchymal cells. Particularly CD47 decrease moderately and PD‐L1 was not detectable in the mesenchymal cells. Overall, these data demonstrated that the expressions of septin 9 and ‘don't eat me’ signal molecules increased in epithelial like CCA cells and were inverse correlated to the EMT process.

### 3.7 Septin 9 regulates expression of EMT markers in CCA cells

**Fig. 6:**
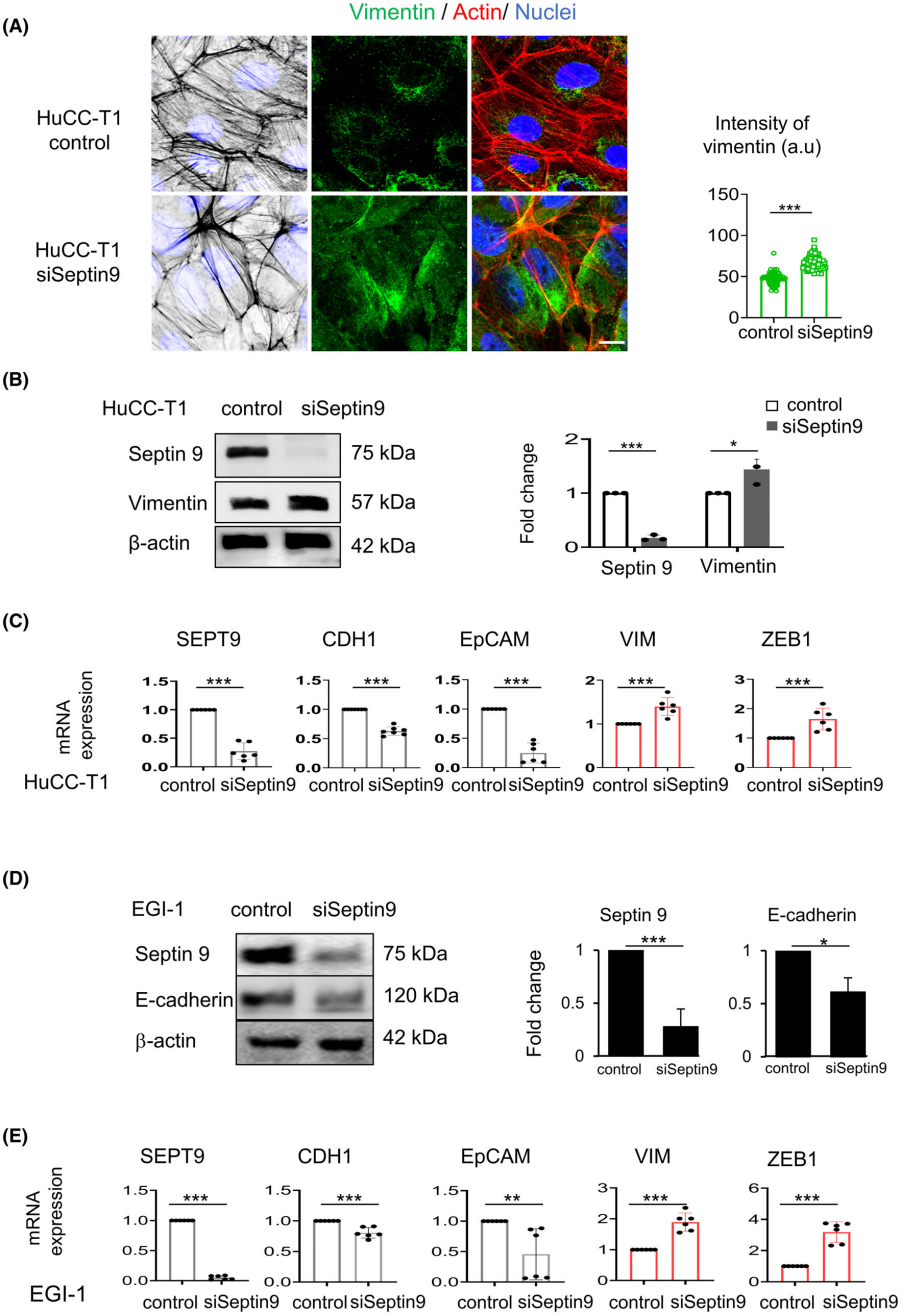
Septin 9 regulates expression of mesenchymal markers in iCCA cells. (A) HuCC‐T1 cells were grown for 24 h and treated with siSeptin 9 or control for 48 h, and then stained for vimentin (green) and actin (red) before analysis using confocal microscopy: scale bar, 10 μm, *n* = 3. The intensity of vimentin is presented as beside (***P < 0.001, Student's *t*‐test). (B) HuCC‐T1 cells were treated like (A), then cell lysates were analyzed by immunoblot for septin 9 and vimentin. β‐actin was used as a loading control, *n* = 3. The fold change of the proteins was shown to the right (*P < 0.05, ***P < 0.001, Student's *t*‐test). (C) With the same condition as (A), the HuCC‐T1 cells were analyzed for *SEPT9*, *CDH1*, *EpCAM*, *VIM*, and *ZEB1* mRNA expression levels by RT‐qPCR. The bar graphs showed the results, *n* = 3 (***P < 0.001, Student's *t*‐test). (D) EGI‐1 cells were treated like (A), then cell lysates were analyzed by immunoblot for septin 9 and E‐cadherin. β‐actin was used as a loading control. The fold change of the proteins was shown to the right *n* = 3 (*P < 0.05, ***P < 0.001, Student's *t*‐test). (E) With the same condition as (D), the EGI‐1 cells were analyzed for *SEPT9*, *CDH1*, *EpCAM*, *VIM*, and *ZEB1* mRNA expression levels by RT‐qPCR, *n* = 3. The bar graphs showed the results (**P < 0.01, ***P < 0.001, Student's *t*‐test). All data represented mean ± SEM (Student's *t*‐test). (iCCA, Intrahepatic cholangiocarcinoma; RT‐qPCR, reverse transcription and real‐time PCR analysis; *VIM*, vimentin gene; *CDH1*, E‐cadherin gene; EpCAM, epithelial cell adhesion molecule; siSeptin9, siRNA target *SEPT9*; *SEPT9*, septin 9 gene).

We recently reported that knock‐down of septin 9 using siRNA or deletion of its polybasic domains (PBs) increased the expression of the mesenchymal markers [38]. Thus, to determine whether septin 9 controls EMT in CCA cells, we knocked down septin 9 in HuCC‐T1 cells using specific siRNA and control cells were treated with scramble siRNA. Immunofluorescence analysis revealed a significant increase of vimentin staining in cells treated with siRNA compared to control cells (Fig. 6A) and these data were confirmed by immunoblot (Fig. 6B, Fig. S10A). Furthermore, we performed RT‐qPCR, and we found a decreased mRNA of E‐cadherin and EpCAM while those of vimentin and ZEB‐1 significantly increased (Fig. 6C). Subsequently, we knocked down septin 9 in EGI‐1 cells and performed RT‐qPCR and immunoblot. The results confirmed the effects of septin 9 on EMT markers (Fig. 6D,E). Together these data indicated that septin 9 regulated EMT in CCA cells.

### 3.8 Septin 9 expression is regulated by IFN‐γ and controls the ‘don't eat me’ signal in CCA cells

**Fig. 7:**
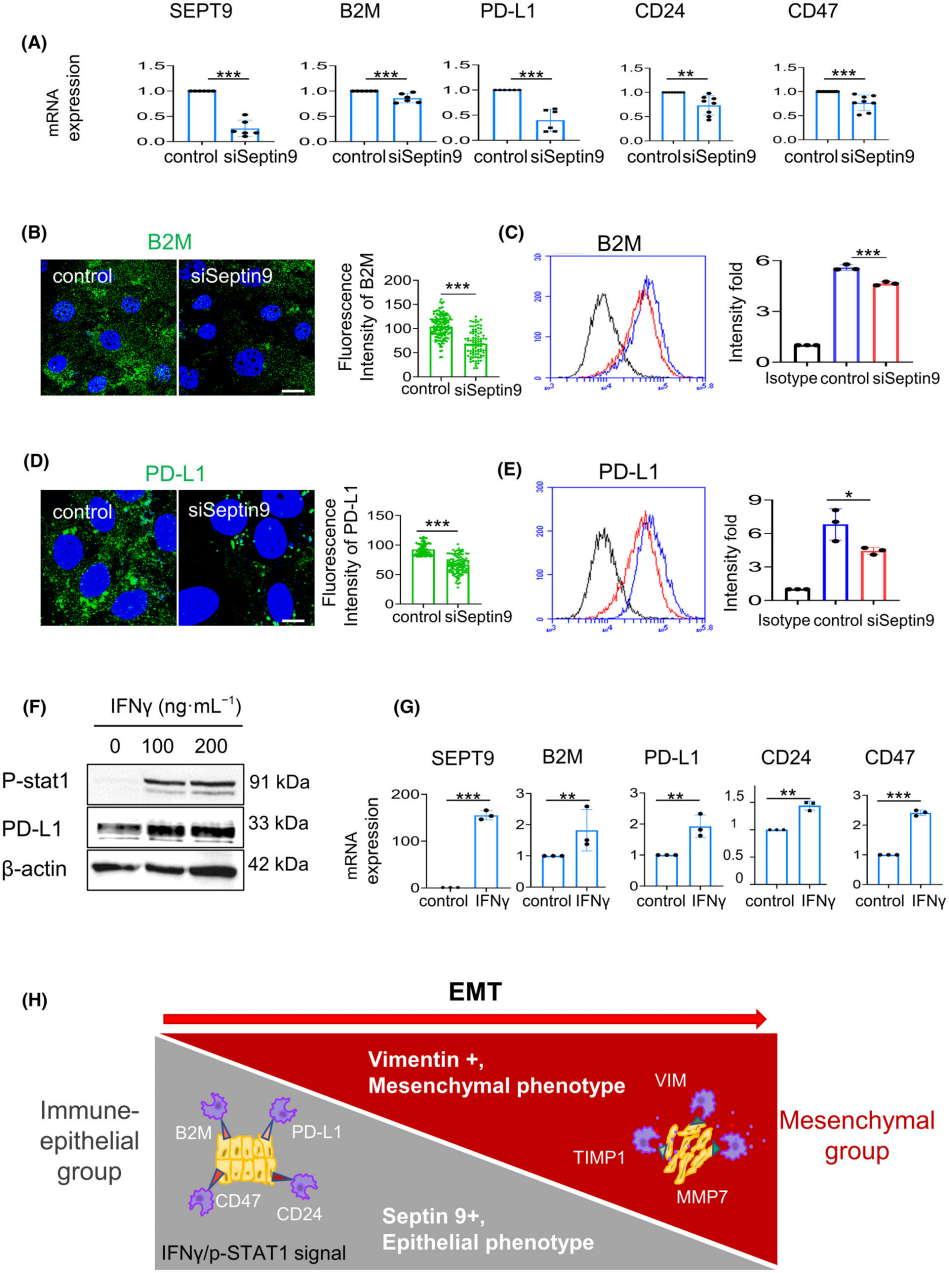
Septin 9 regulates expression of the epithelial‐immune markers in iCCA cells. (A) HuCC‐T1 cells were grown for 24 h and treated with siSeptin 9 or control for 48 h. Then, the cells were analyzed for *SEPT9*, and ‘don't eat me’ genes mRNA expression by RT‐qPCR (**P < 0.01, ***P < 0.001, Student's *t*‐test), *n* = 3. (B, D) HuCC‐T1 cells transfected as in (A) were fixed and then stained for B2M (green) or PD‐L1 (green) before analysis using confocal microscopy: scale bar, 10 μm, *n* = 3. Next, the quantification of the fluorescence intensity of PD‐L1 or B2M was represented as a histogram beside the image (***P < 0.001, Student's *t*‐test), *n* = 3. (C, E) Cells transfected or not with siSeptin9, were stained for B2M or PD‐L1 surface expression, *n* = 3. The fluorescence intensity was measured by flow cytometry. Representative flow data from three independent experiments are presented as a graph, *n* = 3. The quantification of the fluorescence intensity fold of B2M or PD‐L1 from different conditions is represented as a histogram (*P < 0.05, ***P < 0.001, Student's *t*‐test). (F) HuCC‐T1 cells were grown for 24 h and then stimulated with IFN‐ γ in 100 and 200 ng·mL^−1^ for 24 h, and the cell lysates were analyzed by immunoblot for pSTAT1 and PD‐L1. β‐actin was used as a loading control. The data from a preliminary experiment, *n* = 2. (G) With the 100 ng·mL^−1^ IFN‐ γ treatment as (F), HuCC‐T1 cells were collected for *SEPT9* and ‘don't eat me’ genes mRNA expression by RT‐qPCR (**P < 0.01, ***P < 0.001, Student's *t*‐test), *n* = 3. (H) The schematic graph shows that cytoskeleton septin 9 and vimentin can divide CCA cells into two groups. We termed the septin 9 high expression the ‘Immune‐epithelial group,’ which was featured as epithelial morphology and expressed epithelial markers and the ‘don't eat me’ signal. The IFN‐ γ/p‐STAT1 signal regulated the septin 9 and ‘don't eat me’ signal. The other with high vimentin mesenchymal morphology termed the ‘Mesenchymal group’, contained TIMP1 and MMP7 and mesenchymal markers. All data represented mean ± SEM (Student's *t*‐test). (iCCA, Intrahepatic cholangiocarcinoma; RT‐qPCR, Reverse Transcription and Real‐time PCR analysis; PD‐L1, programmed cell death ligand 1; B2M, β‐2 macroglobulin subunit of the major histocompatibility class I complex; pSTAT1, phosphorylation of STAT1; IFN‐γ, interferon‐γ; siSeptin9, siRNA target *SEPT9*).

To seek for the contribution of septin 9 in the ‘don't eat me’ signals, HuCC‐T1 cells were treated with septin 9 siRNA, allowing a substantial decrease of septin 9 transcript and also that of B2M, PD‐L1, CD24 and CD47 (Fig. 7A). We further investigated the effects of septin 9 on the decrease of B2M and PD‐L1 expressions using immunofluorescence (Fig. 7B,D) and flow cytometry (Fig. 7C,E).

IFN‐γ is a cytokine critical for innate and adaptative immunity and functions as the primary activator of macrophages and enhances immune recognition of cancer cells. In response to IFN‐γ stimulation, STAT1 is phosphorylated, then dimerized and translocated into the nucleus to activate transcription, and thus promoting the cellular activity of IFN‐γ. IFN‐γ stimulates the expression of a group of immune genes, including PD‐L1 [49], B2M [50], and CD47 [51], that impacts the effectiveness of anti‐cancer immune surveillance. Therefore, we hypothesized that IFN‐γ signal might regulate *SEPT9* as reported for the ‘don't eat me’ genes. Subsequently, HuCC‐T1 cells were treated with IFN‐γ at 100 and 200 ng·mL^−1^ for 24 h, and we assessed the phosphorylation of STAT1 (pSTAT1) which strikingly increased (Fig. 7F, Fig. S10B). Furthermore, we observed that *SEPT9* dramatically increased with IFN‐γ stimulation, as well as the ‘don't eat me’ genes (Fig. 7G). Thus, these data strongly suggested that *SEPT9* is an element of the IFN‐γ signal that regulates the ‘don't eat me’ signal.

### 3.9 Septin 9 and PD‐L1 expressions are positively correlated in CCA tissues and are associated with restrict tumor infiltrated lymphocytes (TIL)

**Fig. 8:**
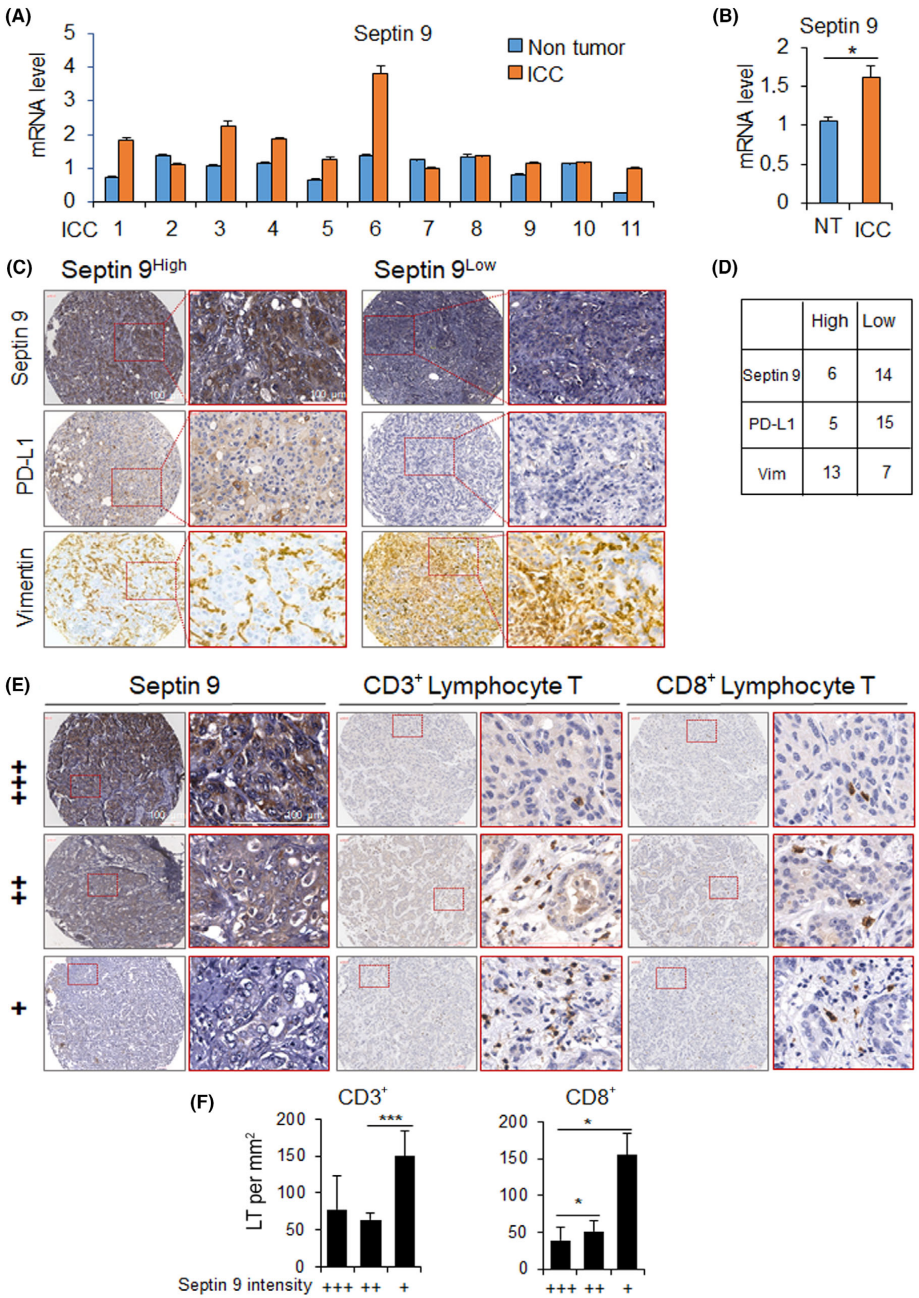
Septin 9 and PD‐L1 expression in iCCA tissues. (A) Analysis of the mRNA levels of septin 9 by RT‐qPCR for 11 samples of iCCA with corresponding NT (non‐tumor) area. (B) The mean of analysis in (A) Values presented as mean ± SEM (*P < 0.05, Student's *t*‐test). (C) Immunohistochemistry staining of septin 9, PD‐L1 and vimentin in 20 samples iCCA on a TMA (Tissue Microarray). Negative, weak (+), moderate (++), and strong‐level expression (+++) were the classifications assigned to specimens with total scores (as described in Section 2). Among these, group Low comprises negative and weak expression, whereas group High comprises moderate and high expression. Typical staining outcomes of high/low septin 9 are presented, juxtaposed with the PD‐L1 and vimentin staining using the identical sample. Scale bar, 100 μm. (D) Count of samples exhibiting low/high levels of Septin 9, PD‐L1 and vimentin staining. (E) Immunohistochemistry staining of CD3 and CD8 in 20 samples iCCA on the same TMA for septin 9. The typical staining results of septin 9, showcasing three distinct intensity levels as mentioned in (C), are presented alongside CD3/CD8 staining using the same sample. Scale bar, 100 μm. (F) Expression correlation studies of infiltrating CD3^+^/CD8^+^ T lymphocyte with septin 9. Groups: weak (+) *n* = 14; moderate (++) *n* = 3; strong (+++) *n* = 3. Lymphocyte counts were quantified in three randomly selected areas of 0.11 square millimeters each per sample. Values presented as mean ± SEM (*P < 0.05, ***P < 0.001, Student's *t*‐test). (iCCA, Intrahepatic cholangiocarcinoma; LT, lymphocyte T cells; NT, non‐tumor; PD‐L1, programmed cell death ligand 1; RT‐qPCR, Reverse Transcription and Real‐time PCR analysis; TMA, tissue microarray).

To validate the transcriptomic and experimental data, we performed investigations using clinical iCCA samples. First, we demonstrated that the expression of septin 9 increased in iCCA compared to the adjacent non‐tumor area using RT‐qPCR (Fig. 8A,B). Therefore, we analyzed septin 9, PD‐L1 and vimentin by immunohistochemistry staining of iCCA samples on Tissue Microarray (TMA). Among the 20 samples analyzed, the expression of septin 9 and PD‐L1 were very similar. Indeed, a ratio of 6/14 for high/low expression was obtained for septin 9 and 5/15 for PD‐L1 (Fig. 8C,D), thus revealed a positive relation between septin 9 and PD‐L1 in iCCA. The expression of vimentin is opposite to that of both septin 9 and PD‐L1 (Fig. 8C) in line with our experimental data using CCA cell lines (Fig. 5).

Tumor infiltrating lymphocytes are often associated with good prognosis in cancer patients [52, 53, 54, 55]. To further investigate the relation between septin 9 and immune system, we stained for CD3 and CD8 lymphocytes in iCCA using immunohistochemistry on the TMA used to analyze septin 9 and PD‐L1 (Fig. 8C). A typical staining of CD3 and CD8 lymphocytes on iCCA samples with different intensities of septin 9 expression were presented (Fig. 8E). Analysis of the data revealed a low density of TILs when septin 9 expression was low (septin 9+), while their number significantly increased in samples with high septin 9 expression (septin 9+++) (Fig. 8F). A similar result was observed with CD8 staining (Fig. 8E,F). Together these data revealed an inverse correlation between septin 9 expression and TILs in iCCA, promoting tumor evasion from immune system.

## 4 Discussion

The incidence of iCCA is increasing worldwide while there are few effective treatments. Immunotherapy opens new oncology treatment perspectives, while its efficacy and safety remain unclear in iCCA. There is a need to identify reliable predictive biomarkers for immunotherapy to be more effective in iCCA.

In this study, we investigated septin 9 and vimentin as potential biomarkers of iCCA. Based on the single‐cell transcriptome data, we showed that septin 9 was enriched in iCCA cells and low in HCC. Its expression was higher in HPC‐like than malignant cells and was positively correlated with the expression of CK19. Interestingly, *in vitro* experiments and data from the CCLE transcriptome database confirmed that septin 9 is higher in CCA cells which exhibited epithelial‐like features compared to vimentin‐enriched mesenchymal‐like cells. Interestingly septin 9 also increased in eCCA cells, suggesting its broader role in the heterogeneity of CCA. Therefore, we could speculate that septin 9 represents a cytoskeletal marker of epithelial cells as vimentin is for mesenchymal cells.

Notably, the analysis of septin 9 and vimentin expressions in iCCA cells allowed us to characterize two clusters of iCCA cells as recapitulated in Fig. 7H. One of these clusters were enriched in vimentin and ECM remodeling molecules, such as TIMP1 and MMP7, and is named the ‘mesenchymal‐ECM group’, representing cells with invasive features. Importantly B2M, a member of the ‘don't eat me’ signaling genes, was highly expressed in septin 9‐enriched clusters, exhibiting an unprecedented description of B2M in iCCA cells. Furthermore, we showed the cellular distribution of septin 9 and CD24, and CD47, two other members of the ‘don't eat me’ signal. Accordingly, we named this cluster the ‘immune‐epithelial group’.

Interestingly, we demonstrated a positive correlation between septin 9 expression and the ‘don't eat me’ signal genes and the knockdown of septin 9 decreased the ‘don't eat me’ signal genes. Furthermore, we showed that septin 9 expression increased upon stimulation of CCA cells by IFN‐γ as the other ‘don't eat me’ signal genes. Therefore, we conclude that by regulating the expression of these ‘don't eat me’ signal genes, septin 9 could control the escape of the epithelial‐ like CCA cells from phagocytosis of macrophage and natural killer (NK) cells to maintain their survival. Importantly, septin 9 isoform 1 (*SEPT9_i1*) protein associates with hypoxia‐inducible factor (HIF)‐1α to increase HIF‐1 transcriptional activity. HIF‐1α interacts specifically to importin‐α7 which mediated its nuclear translocation [56]. PD‐L1 is recognized as a direct target of HIF‐1α and the different reports revealed that HIF‐1α binds to the promoter region of PD‐L1 and regulated its expression [57]. According to these data, we proposed that septin 9 could also regulate PD‐L1 and CD47 expressions by regulating HIF‐1α.

By contrast to the ‘don't eat me’ signals, ‘eat me’ signals are specifically displayed on the surface of apoptotic cells for selective removal by phagocytosis [58]. Vimentin was described as a receptor for ‘eat‐me’ signals that facilitated both professional and non‐professional phagocytosis of apoptotic cells and promoted immune attack [11, 59]. Thus, the mesenchymal‐like cells enriched in vimentin may be more susceptible to immune attack from macrophages. Notably, the phagocytic capacity is increased by cleavage of membrane type 6 matrix metalloproteinase (MT6‐MMP), a neutrophil‐specific protease [59]. Among the members of the TIMPs family, natural inhibitors of matrix metalloproteinases, TIMP1 is a more effective inhibitor of MT6‐MMP [60]. TIMP1 is present in the vimentin‐enriched ‘mesenchymal group’. Furthermore, recent reports on lung cancer, demonstrated that EMT increased the sensitivity of cancer cells to be destroyed by NK cells and, to some extent, reduced tumor metastasis, thus proposing a new concept of NK‐mediated immune surveillance [61]. All these data somehow support the idea that the mesenchymal‐like cells in iCCA, are sensitive to immune attack and reinforced our data and hypotheses.

However, by contrast to these data described above, several articles reported a positive correlation between PD‐L1 and vimentin expressions in cancer were reported [62]. Vimentin is classical marker of EMT and here again recent reports described EMT as a process that induces immune escape of cancer cells [63] and increases PD‐L1 expression [64]. EMT is a complex process and is not sufficient to study by assessing several markers. The methods to assess in one side EMT and in the other side, PD‐L1 which could be expressed in both cancer cells and tumor micro‐environment cells could strongly impact the interpretation of the relation between EMT and PD‐L1 expression. Moreover, there is an important intra‐ and inter‐ tumor heterogeneity in cancer and all these points could be at the basis of these difference in the published results. Therefore, our study here, based on single cell or cell line analyses appeared as an excellent strategy to decipher the relation between cancer cell intrinsic expression of PD‐L1 and the EMT process.

Eventually, EMT is often associated with a dense stroma as observed in iCCA and the stiffness of ECM represents another barrier, which could block the recruitment of immune cells into the tumor. Thus, representing another mechanism of resistance of cancer cells to the immune system. Nevertheless, the mechanism involved in immune escape and the relation with EMT need further investigation. In this context, the study of septin 9 might represent a novel way to understand the relationship between EMT and immune response.

Immunotherapy opens new prospects for better prevention of tumor development and metastasis. PD‐L1 is expressed and serves as a therapeutic target for immune‐checkpoint inhibitors in many cancers. Furthermore, monoclonal antibodies against the ‘don't eat me’ signal and their receptors expressed by macrophages have been shown to have therapeutic potential for some cancers [25]. Likewise, anti‐CD47 has been shown to promote the phagocytosis of macrophages, thereby inhibiting the growth and metastasis of iCCA. Overall, septin 9 appears to be a promising biomarker that could be used to select CCA patients to improve response to immunotherapy, especially against PD‐L1 therapies.

## 5 Conclusion

In this study, we identified septin 9 as an EMT and host immune response regulator. Combining vimentin and ‘don't eat me’ gene with septin 9 can further classify CCA precisely, allowing the selection of cancers sensitive to immunotherapy.

## Conflict of interest

The authors declare no conflict of interest.

## Author contributions

TtC performed experiments on the characterization of the ‘don't eat me’ signal genes, contributed to EMT studies, prepared the figures, wrote the related comments and discussion, and edited the manuscript. CD designed and performed the bioinformatic studies based on single‐cell transcriptomic data, wrote related comments, and edited the manuscript. JP performed experiments with CCA cells, immunohistochemical data analyses and wrote related comments. JA performed bioinformatic analysis on the CCLE database. PS performed a RT‐qPCR‐based analysis of EMT using CCA cells. DO performed experiments with CCA cells. ADS and CG performed the immunohistochemical analyses. DS contributed to interpretation and discussion of the data. AG‐D conceived, designed, and supervised the study and wrote the manuscript.

### Peer review

The peer review history for this article is available at https://www.webofscience.com/api/gateway/wos/peer‐review/10.1002/1878‐0261.13673.

## Supporting information


**Fig. S1.** The landscape of liver tumor heterogeneity.
**Fig. S2.** *CK19*, *CDH1* and other *SEPTs* expression in cells from liver tumors.
**Fig. S3.** Double positive *CK19‐CDH1* population in iCCA tumor cells.
**Fig. S4.** Pseudotime transformation according to alternative expression of *SEPT9* and *VIM* in iCCA tumor cells.
**Fig. S5.** Pseudotime expression *CK19*, *EpCAM* and *CDH1* in iCCA tumor cell compartment.
**Fig. S6.** Interface of the interactive web application.
**Fig. S7.** Expression of markers following *VIM* and *SEPT9* cell decision in iCCA tumor cells.
**Fig. S8.** Function analysis of *VIM* and vim clusters single cell in iCCA.
**Fig. S9.** Septin 9 and vimentin expression patterns characterize epithelial‐immune and mesenchymal iCCA cells.
**Fig. S10.** The raw data for the immunoblotting bands in the figures.
**Table S1.** The primers sequences of the genes tested in the experiment.
**Table S2.** Best one hundred markers found on branching 2 of the pseudotime transformation based on alternative expression of septin 9‐vimentin in intrahepatic cholangiocarcinoma tumor cells.

## Data Availability

The authors confirmed that the data supporting the findings of the study are available in the article and Supplementary materials. The single‐cell analyses are available on an internet interface was developed at the following address: https://hsce.shinyapps.io/icca/. Any additional information required to re‐analyze the data reported in this article is available from the lead contact upon request.
