# Supplementary material for: Septin 9 expression regulates ‘don't eat me’ signals and identifies an immune–epithelial class of intrahepatic cholangiocarcinoma
Source: Mol Oncol. 2024 Jul 31;18(10):2369–92. doi: 10.1002/1878-0261.13673 (PMC11459040; doi:10.1002/1878-0261.13673)
Supplement: Supplementary file 1 — Fig. S1. The landscape of liver tumor heterogeneity. Fig. S2. CK19, CDH1 and other SEPTs expression in cells from liver tumors. Fig. S3. Double positive CK19‐CDH1 population in iCCA tumor cells. Fig. S4. Pseudotime transformation according to alternative expression of SEPT9 and VIM in iCCA tumor cells. Fig. S5. Pseudotime expression CK19, EpCAM and CDH1 in iCCA tumor cell compartment. Fig. S6. Interface of the interactive web application. Fig. S7. Expression of markers following VIM and SEPT9 cell decision in iCCA tumor cells. Fig. S8. Function analysis of VIM and vim clusters single cell in iCCA. Fig. S9. Septin 9 and vimentin expression patterns characterize epithelial‐immune and mesenchymal iCCA cells. Fig. S10. The raw data for the immunoblotting bands in the figures. Table S1. The primers sequences of the genes tested in the experiment. Table S2. Best one hundred markers found on branching 2 of the pseudotime transformation based on alternative expression of septin 9‐vimentin in intrahepatic cholangiocarcinoma tumor cells. [file MOL2-18-2369-s001.zip › aSupplemental material and methods.pdf]

Supplemental Figures information

Figure S1. The landscape of liver tumor heterogeneity. Cells were rearranged based on the known lineage-specific marker genes as cancer-associated fibroblasts (CAFs), tumor-associated endothelial cells (TECs), T cells, B cells, tumor-associated macrophages (TAMs), hepatic progenitor cell markers (HPC-like), malignant cells, and unclassified cells.

Figure S2. *CK19, CDH1* and other *SEPTs* expression in cells from liver tumors. (**a**) t-SNE dimplot of *CK19* expression in cells from liver tumors stratified by their origins HCC and iCCA. (**b**) t-SNE dimplot of *CDH1* expression in cells from liver tumors stratified by their origins HCC and iCCA. (**c**) t-SNE dimplot of *SEPT7* expression in cells from liver tumors stratified by their origins HCC and iCCA. (**d**) t-SNE dimplot of *SEPT2* expression in cells from liver tumors stratified by their origins HCC and iCCA.

Figure S3. Double positive *CK19-CDH1* population in iCCA tumor cells. (**a**) t-SNE dimplot of *CDH1* expression in iCCA tumor cells stratified by their phenotypes. (**b**) Scatterplot of *CK19* versus *CDH1* expressions in iCCA tumor cells stratified on their phenotypes.

Figure S4. Pseudotime transformation according to alternative expression of *SEPT9* and *VIM* in iCCA tumor cells. (**a**) Tree resulting of the single cell transcriptome pseudotime transformation concerning iCCA tumor cells. (**b**) Pseudotime transformation of the single cell data according to the cell group definition (distinct colors) based on alternative expression of septin 9 and vimentin in iCCA tumor cells.

Figure S5. Pseudotime expression *CK19, EpCAM* and *CDH1* in iCCA tumor cell compartment. (**a**/**c**/**e**) Pseudotime tree with *CK19/EpCAM/CDH1* expression as dot size. (**b**/**d**/**f**) Pseudotime expression of *CK19/EpCAM/CDH1* for *VIM* and *SEPT9* distinct subpopulations in iCCA tumor cells.

Figure S6. Interface of the interactive web application. (**a**) Web address of the website with the corresponding QR-code. (**b**) Screenshot with usage steps of the web application which resume expression of heterogeneity which follow cell trajectory of the *SEPT9-VIM* alternative expression in iCCA tumor cells.

Figure S7. Expression of markers following *VIM* and *SEPT9* cell decision in iCCA tumor cells. (**a**/**c**/**e**) t-SNE dimplot of *VIM/TIMP1/MMP7* expression in iCCA tumor cells. Pseudotime tree with *VIM/TIMP1/MMP7* expression as dot size. Pseudotime expression of *VIM/TIMP1/MMP7* for *VIM* and *SEPT9* distinct subpopulations in iCCA tumor cells. (**b**/**d**/**f**) t-SNE dimplot of *SEPT9/CD74/B2M* expression in iCCA tumor cells. Pseudotime tree with *SEPT9/CD74/B2M* expression as dot size. Pseudotime expression of *SEPT 9/CD74/B2M* for *VIM* and *SEPT9* distinct subpopulations in iCCA tumor cells.

Figure S8. Function analysis of *VIM* and *SEPT9* clusters single cell in iCCA. (**a-b**) The top 10 cellular biological processes using GO database enrichment analysis of *VIM* and *SEPT9* clusters. (**c-d**) The network enrichment analysis of *VIM* and *SEPT9* cluster.

Figure S9. Septin 9 and vimentin expression patterns characterize epithelial-immune and mesenchymal iCCA cells. (**a**) Based on the CCLE database, we selected a list of iCCA cell lines to see the expression of septin 9 and vimentin. By the PCA analysis of the expression, we classified the cells into three subgroups, *SEPT9+/VIM-*, *SEPT9-/VIM+*, and *SEPT9+/VIM+*. (**b**) Based on the PCA of the CCLE database, the cells were classified into different iCCA subgroups in table. And the quantification of septin 9 and vimentin expression in different groups were presented in bar graph. (**c-d**) The top 10 cellular components related to cell polarity and the top 10 cellular biological processes were annotated with GO enrichment analysis for the *SEPT9+/VIM-* subgroup cells. (**e**) Based on the *SEPT9* and *VIM* expression, the genes related to the processes *B2M*, *CD74*, *IRF6*, *IRF1*, *STAT2*, *IRF5*, and *IRF8* were presented (*$p<0.05$, **$p<0.01$, t-test).

Figure S10. The raw data for the immunoblotting bands in the figures. (**a**) The raw data for the immunoblotting bands in the Figure 6b for HuCCT1 cells treated with siseptin9 or control. (**b**) The raw data for the immunoblotting bands in the Figure 7f for HuCCT1 cells treated with 0 IFN γ in 0, 100, 200 ng/ml.

Table S1: **The primers sequences of the genes tested in the experiment**

Primer name	Forward sequence	Reverse sequence
SEPT9	GTC CAC TGC TGC CTC TAC TTC A	GGA CGA TGT TGA CCA CCT TGC T
VIM	CCC TGC AAT CTT TCA GAC AG	ATC TGA GCC TGC AGC TCC T
CDH1	AAG AAG CTG GCT GAC ATG TAC GGA	CCA CCA GCA ACG TGA TTT CTG CAT
EpCAM	CCA TGT GCT GGT GTG TGA A	TGT GTT TTA GTT CAA TGA TGA TCC A
ZEB1	GCT GTT GTT CTG CCA ACA GTT GGT T	CCA CCT TGT TGT ATG GGT GAA GCA
SNAIL	TGC CCT CCA GAT GCA CAT CCG A	GGG ACA GGA GAA GGG CTT CTC
B2M	CCA CTG AAA AAG ATG AGT ATG CCT	CCA ATC CAA ATG CGG CAT CTT CA
PD-L1	TGC CGA CTA CAA GCG AAT TAC TG	CTG CTT GTC CAG ATG ACT TCG G
CD24	CAA TAT TAA ATC TGC TGG AGT TTC ATG	TCC ATA TTT CTC AAG CCA CAT TCA
CD47	AGA AGG TGA AAC GAT CAT CGA GC	CTC ATC CAT ACC ACC GGA TCT
GAPDH	AGC CAC ATC GCT CAG ACA C	GCC CAA TAC GAC CAA ATC C

Table S2. Best one hundred markers found on branching 2 of the pseudotime transformation based on alternative expression of septin 9-vimentin in intrahepatic cholangiocarcinoma tumor cells

gene_short_name	Branching 2 p-values	branching 2 q-values
MTRNR2L12	0	0
MTRNR2L8	0	0
KRT17	1.22583112114659E-287	8.37446960929978E-284
MTRNR2L10	4.23228099552872E-274	2.16851497508403E-270
MTRNR2L1	1.14145542450809E-205	4.67882578505867E-202
MT-ND2	1.25635070315982E-157	4.2914846102101E-154
MT-ND1	2.81801934103091E-157	8.25075805634692E-154
MT-ND5	1.677037519413E-142	4.29636049504619E-139
VIM	9.9059369974481E-132	2.25580198625221E-128
TFF1	9.56091273168847E-130	1.95950906435955E-126
MT-ND4L	9.80476042689437E-128	1.82680513590182E-124
MT-ND4	2.25094823507532E-126	3.84443200648906E-123
GSTP1	1.98567767349479E-122	3.13049722448275E-119
MT-ND3	6.96589166695728E-121	1.0197567836735E-117

MT-CYB	2.08200800489312E-111	2.8447169373523E-108
SERPINA1	4.74906665278883E-109	6.08325756555669E-106
TFF3	2.5337949117416E-107	3.05471333624377E-104
FN1	5.25810734242776E-107	5.98693944350316E-104
FTL	5.14205205870072E-105	5.54665036542481E-102
MMP7	2.20379690397251E-100	2.25834087734583E-97
IFI27	6.06120455035321E-96	5.91544701235662E-93
VCAN	1.05182247366074E-89	9.79868254439862E-87
IGFBP1	7.2891988622476E-84	6.49531002964194E-81
TAGLN	1.52757181953056E-81	1.30448268505328E-78
AKR1B1	2.82291439464595E-77	2.31422522073075E-74
PSCA	1.70864995644288E-73	1.34687618681911E-70
ACTG1	6.92032987924175E-68	5.25304299537258E-65
WFDC2	7.2352593108855E-68	5.29595141344994E-65
NNMT	7.46431526091436E-67	5.27521176801517E-64
RPL41	3.74516604243103E-62	2.5585726013208E-59
AGR2	1.5545549728996E-61	1.02776142482507E-58
B2M	2.76481117742376E-60	1.77077515879062E-57
IGFBP3	1.83853436986288E-59	1.14184127001029E-56
CP	1.65636025384551E-57	9.98444217722462E-55
MT-ATP6	1.95282181663253E-57	1.14351666091096E-54
TFPI	7.59586581896664E-57	4.32436860999226E-54
ATP1B1	1.44483576376861E-55	7.79260762590463E-53
RNASET2	1.43649145095808E-55	7.79260762590463E-53
TIMP1	9.17729180075219E-55	4.82278449888246E-52
TMEM176B	1.88985076503365E-51	9.68312285734118E-49
FGG	1.77686178265348E-50	8.88214200865442E-48
CD74	2.06298273468529E-50	1.00668645588988E-47
OLFM4	6.64874828767923E-50	3.16897898037176E-47
HSP90AA1	1.02192828522506E-49	4.76009550129265E-47
RPS6	2.21609579361597E-49	1.0093085175591E-46
RARRES2	6.61291899375401E-48	2.94634292993453E-45
AC090498.1	1.02924814655298E-47	4.48817888587306E-45

RPL35	1.99797756482227E-46	8.53094795646508E-44
S100P	3.10421390664829E-46	1.29838497993381E-43
RBP1	1.17330259536933E-45	4.80936733841887E-43
RPS10	3.47214106781035E-45	1.39532414087791E-42
POLR2J3	5.91126385530367E-45	2.32983370604709E-42
EIF1	9.92958559142873E-44	3.83975201313834E-41
MALAT1	1.56314886710771E-43	5.93272889469861E-41
S100A9	2.38335005298497E-43	8.88122897016855E-41
ALB	2.72549669988888E-43	9.97483122575403E-41
PDZK1IP1	2.89495293061375E-43	1.04091333882331E-40
S100A2	5.48566174227591E-43	1.9384247828956E-40
ATP5I	7.67989469903917E-43	2.66778715011539E-40
S100A4	9.61715909831909E-43	3.28506126200083E-40
IGFBP2	2.62549565499583E-42	8.82123499166221E-40
COL6A1	3.01114027503543E-42	9.95376128013729E-40
TMSB10	7.60069120424731E-42	2.47263755922299E-39
CD9	3.14574033262505E-41	1.00737418933048E-38
APOE	5.40905575098789E-40	1.70551688640764E-37
MT2A	1.9567604390711E-39	6.07633412102459E-37
TCEB2	5.59174430468458E-39	1.71048954514195E-36
LCN2	1.21709369929236E-38	3.66828461279366E-36
MT-CO2	1.47685428781484E-38	4.38668530851668E-36
RPS26	6.09191301973954E-38	1.78362510485088E-35
KRT23	6.51637242418546E-38	1.88102891315044E-35
PLOD2	7.15818496700436E-38	2.03759723470492E-35
RPL36A	3.03968638768743E-37	8.53402363228135E-35
ARL6IP1	3.36100914079878E-37	9.30863274873931E-35
RP11-14N7.2	3.42967960455444E-37	9.37217113271243E-35
SLC16A3	9.11972428877727E-37	2.45932564866434E-34
FTH1	2.15999511755225E-36	5.74923375769265E-34
MAP1B	1.23029806181503E-35	3.23268702267936E-33
C19orf33	1.95390790993759E-34	5.06903071065454E-32
FAM3C	3.8065031920586E-34	9.75178536515512E-32

GNAS	4.60383441282149E-34	1.16488378136761E-31
LY6E	8.15060898149088E-34	2.03715525702019E-31
RPS20	1.66602807101921E-33	4.11388497777576E-31
GAPDH	2.71206361205029E-33	6.617112348687E-31
FXYD2	3.40636479630838E-33	8.21334664709884E-31
MT1X	5.33250743835818E-33	1.27081092964129E-30
TMEM176A	3.56366613602438E-32	8.39509625951951E-30
CD44	1.35310882335371E-31	3.15135969711752E-29
KRT16	5.65526392068794E-31	1.30229925903932E-28
IGKC	5.78469519110561E-31	1.31730364379677E-28
HLA-B	6.25737012192195E-31	1.40928352361308E-28
TGFBI	6.8666647479162E-31	1.52969884791894E-28
TOB1	1.12917859828223E-29	2.48844251309617E-27
HSD17B11	3.26688047226838E-29	7.1228420509724E-27
ACTN1	4.16777282331048E-29	8.9914214751314E-27
SMIM22	5.78559218775232E-29	1.23516366549983E-26
HLA-DRA	2.21627281633011E-28	4.68273313099851E-26
CCL20	6.8364979138738E-28	1.42973494637595E-25
POLR2L	9.70978216745025E-28	2.0101210658777E-25
KRT6B	1.5075219745007E-27	3.08966628673919E-25
