# Supplementary material for: Septin 9 expression regulates ‘don't eat me’ signals and identifies an immune–epithelial class of intrahepatic cholangiocarcinoma
Source: Mol Oncol. 2024 Jul 31;18(10):2369–92. doi: 10.1002/1878-0261.13673 (PMC11459040; doi:10.1002/1878-0261.13673)
Supplement: Supplementary file 1 — Fig. S1. The landscape of liver tumor heterogeneity. Fig. S2. CK19, CDH1 and other SEPTs expression in cells from liver tumors. Fig. S3. Double positive CK19‐CDH1 population in iCCA tumor cells. Fig. S4. Pseudotime transformation according to alternative expression of SEPT9 and VIM in iCCA tumor cells. Fig. S5. Pseudotime expression CK19, EpCAM and CDH1 in iCCA tumor cell compartment. Fig. S6. Interface of the interactive web application. Fig. S7. Expression of markers following VIM and SEPT9 cell decision in iCCA tumor cells. Fig. S8. Function analysis of VIM and vim clusters single cell in iCCA. Fig. S9. Septin 9 and vimentin expression patterns characterize epithelial‐immune and mesenchymal iCCA cells. Fig. S10. The raw data for the immunoblotting bands in the figures. Table S1. The primers sequences of the genes tested in the experiment. Table S2. Best one hundred markers found on branching 2 of the pseudotime transformation based on alternative expression of septin 9‐vimentin in intrahepatic cholangiocarcinoma tumor cells. [file MOL2-18-2369-s001.zip › Supplemental figures.pdf]

Figure S1

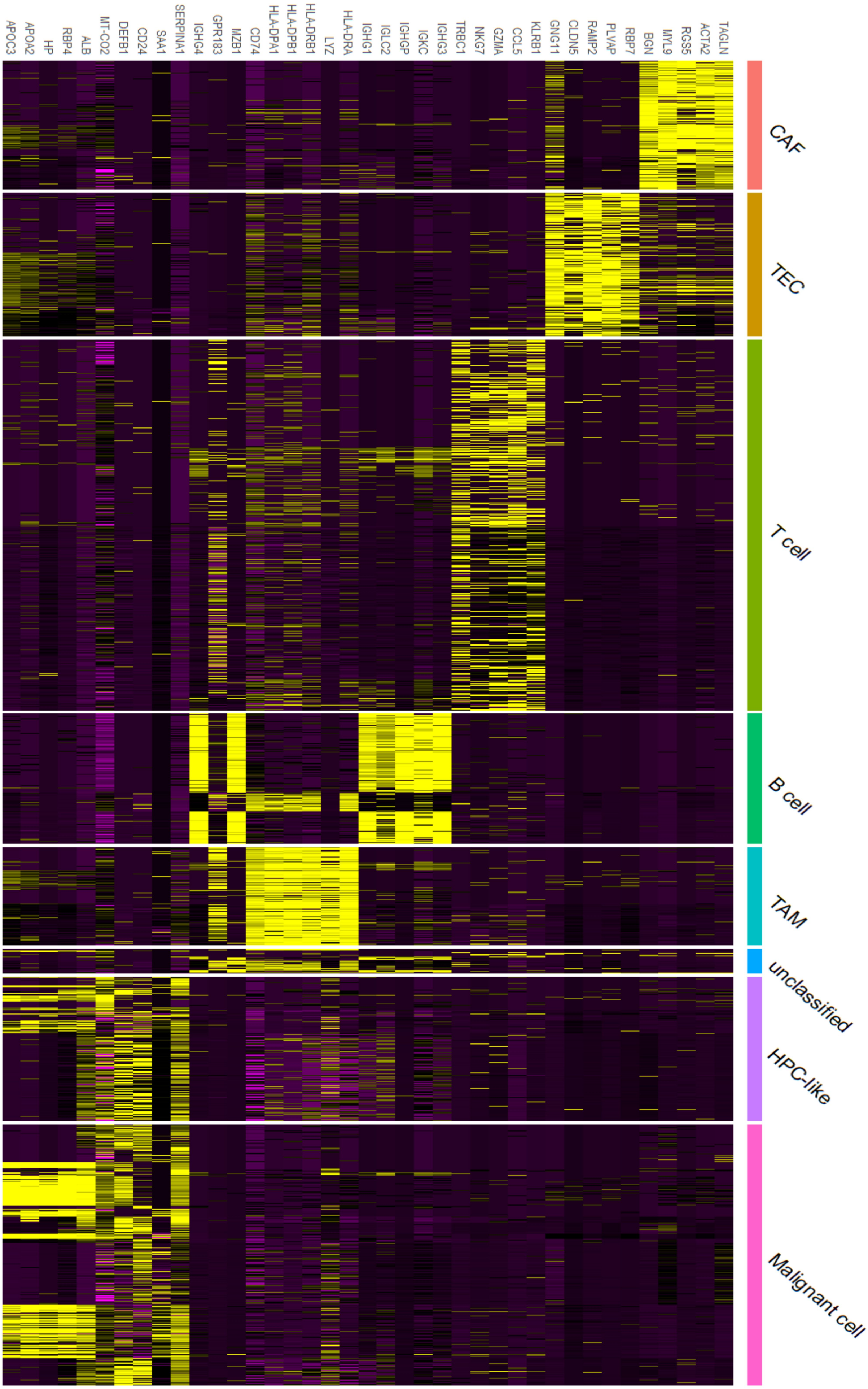
APOC3
APOA2
HP
RBP4
ALB
MT-CO2
DEFB1
CD24
SAA1
SERPINA1
IGHG4
GPR183
MZB1
CD74
HLA-DPA1
HLA-DPB1
HLA-DRB1
LYZ
HLA-DRA
IGHG1
IGLC2
IGHGP
IGKC
IGHG3
TRBC1
NKG7
GZMA
CCL5
KLRB1
GNG11
CLDN5
RAMP2
PLVAP
RBP7
BGN
MYL9
RGS5
ACTA2
TAGLN
CAF
TEC
T cell
B cell
TAM
unclassified
HPC-like
Malignant cell

Figure S2

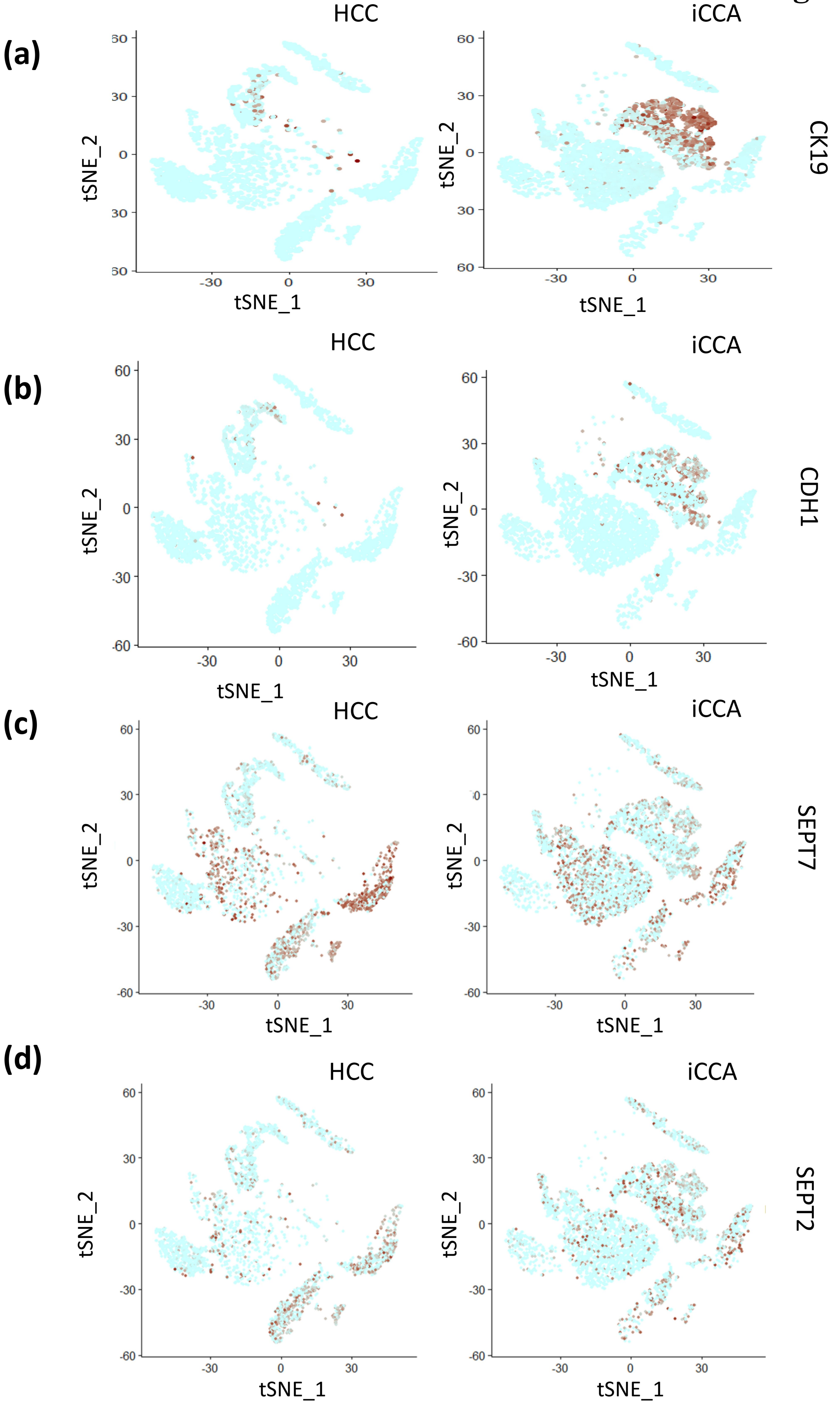
(a)
HCC
iCCA
CK19
(b)
HCC
iCCA
CDH1
(c)
HCC
iCCA
SEPT7
(d)
HCC
iCCA
SEPT2
tSNE_1
tSNE_2

Figure S3

(a)

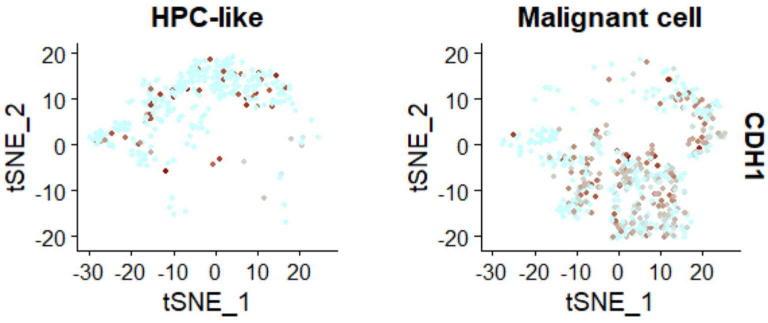
HPC-like
Malignant cell
tSNE_2
tSNE_1
CDH1
20
10
0
-10
-20
-30

(b)

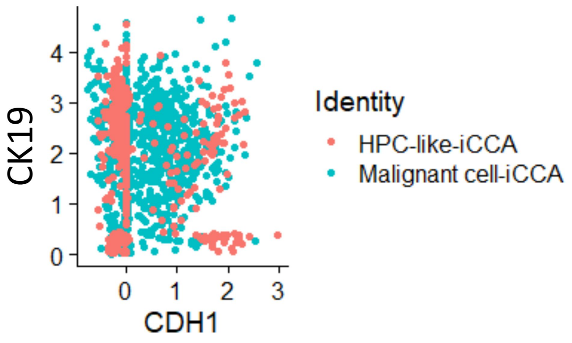
CK19
CDH1
Identity
HPC-like-iCCA
Malignant cell-iCCA

Figure S4

(a)

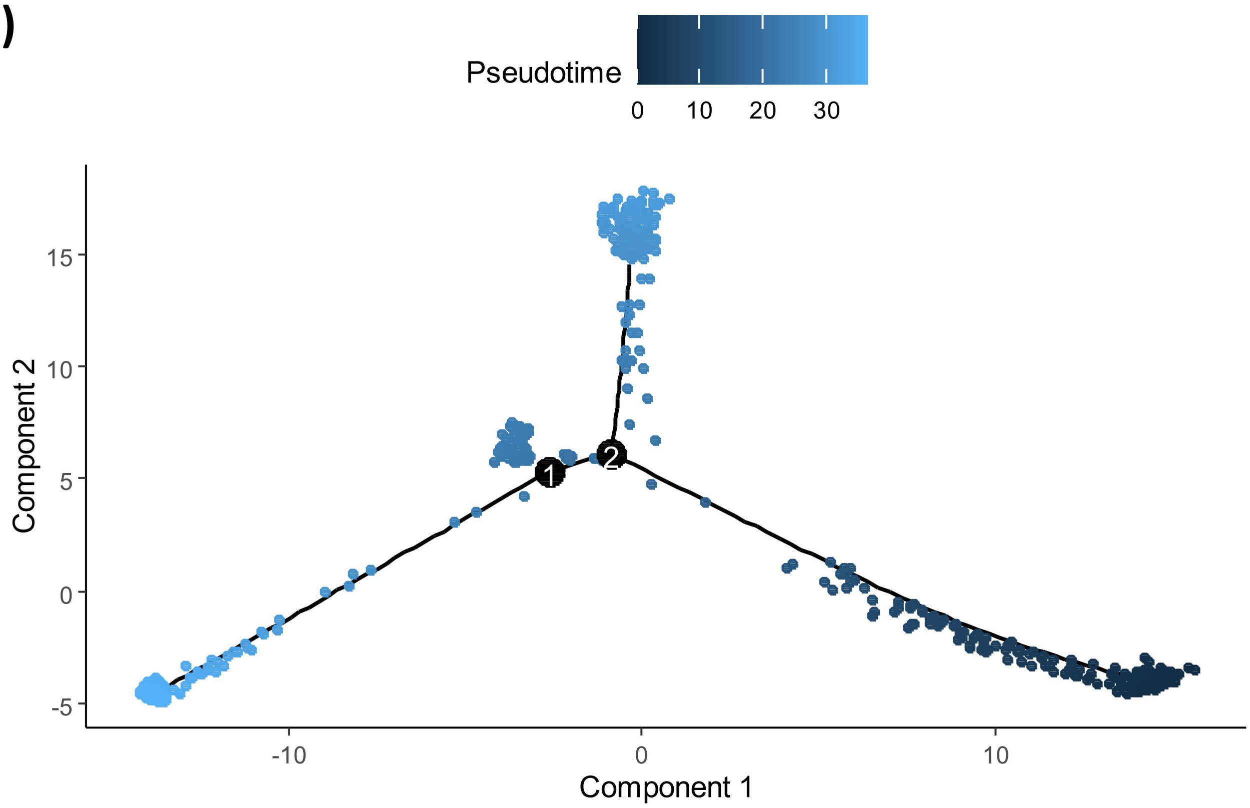

Pseudotime
0
10
20
30
15
10
5
0
-5
Component 2
-10
0
10
Component 1
1
2

(b)

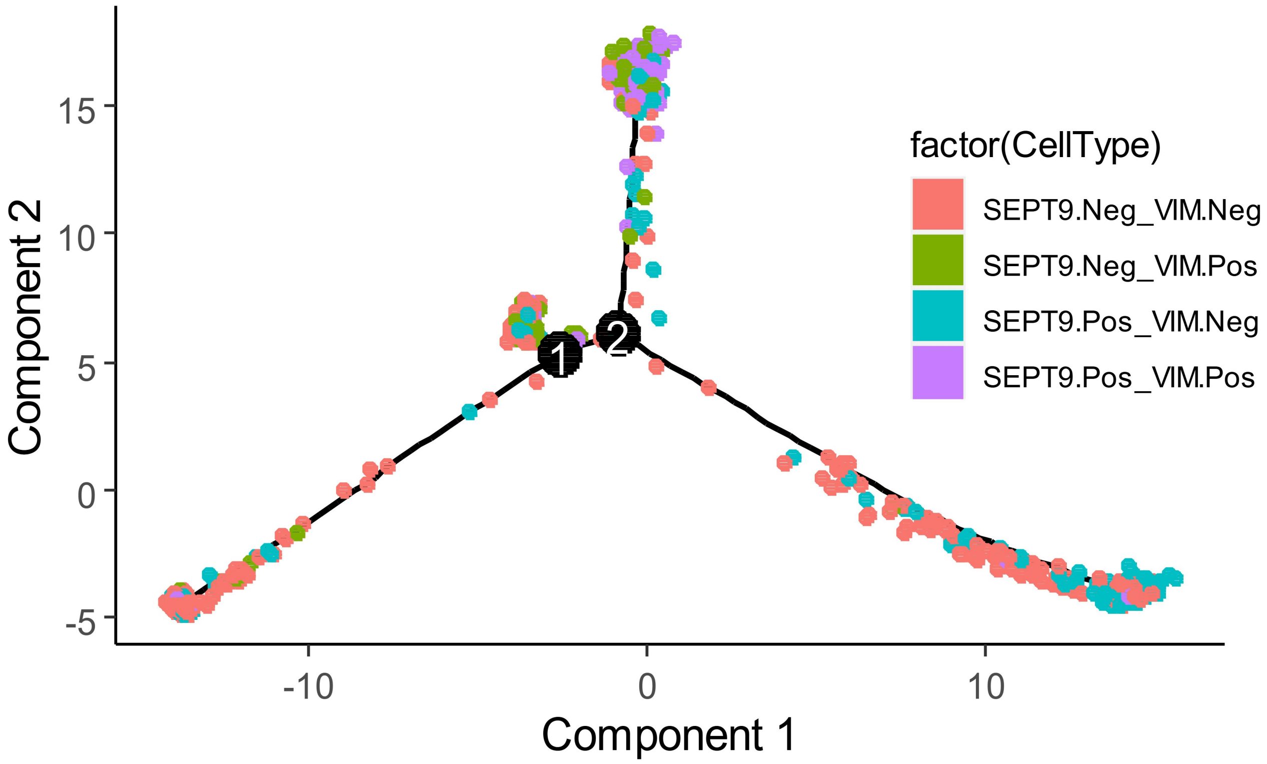

factor(CellType)
SEPT9.Neg_VIM.Neg
SEPT9.Neg_VIM.Pos
SEPT9.Pos_VIM.Neg
SEPT9.Pos_VIM.Pos
15
10
5
0
-5
Component 2
-10
0
10
Component 1
1
2

Figure S5

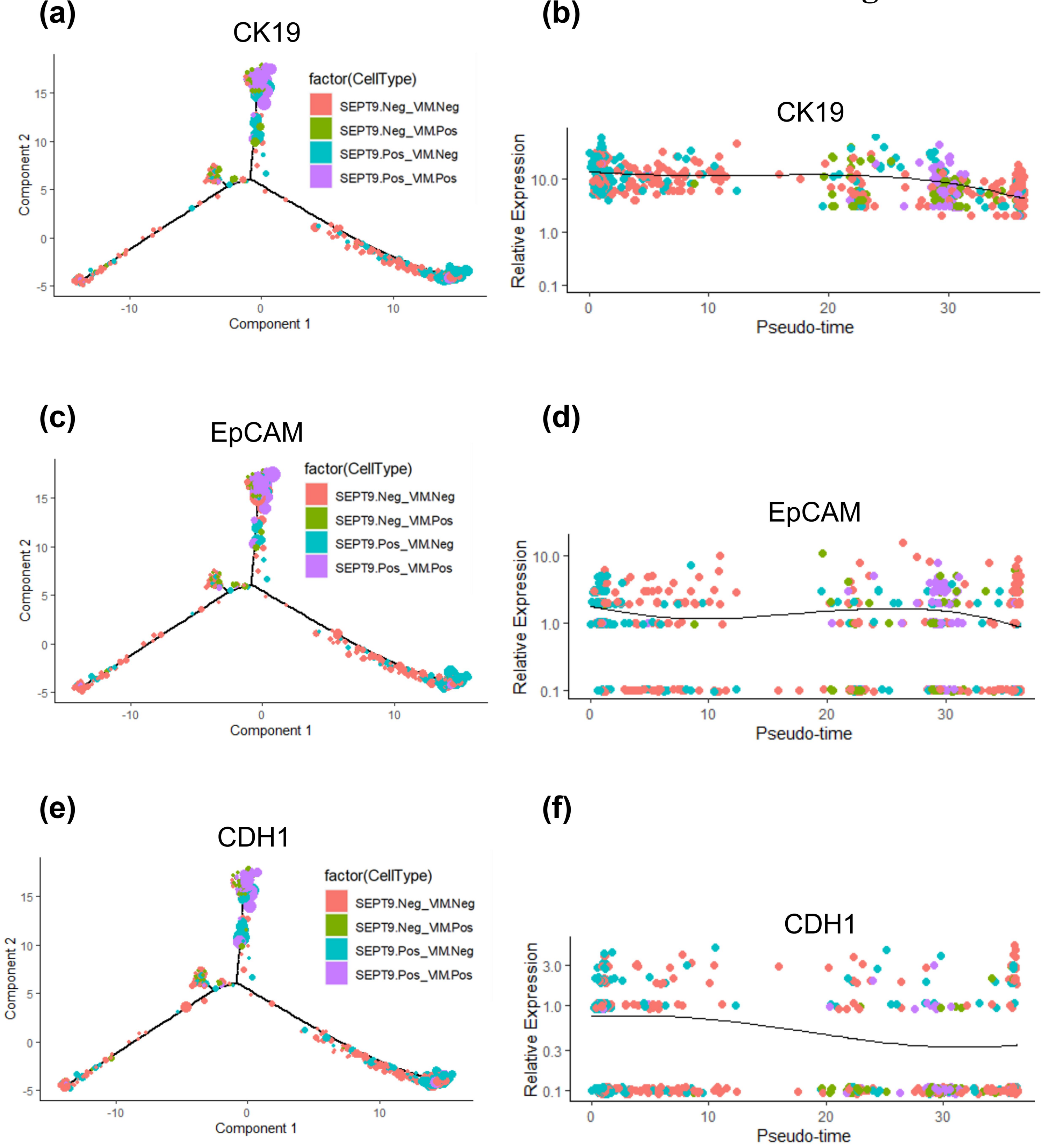
(a)
CK19
factor(CellType)
SEPT9.Neg_VIM.Neg
SEPT9.Neg_VIM.Pos
SEPT9.Pos_VIM.Neg
SEPT9.Pos_VIM.Pos
Component 2
Component 1
(b)
CK19
Relative Expression
Pseudo-time
(c)
EpCAM
factor(CellType)
SEPT9.Neg_VIM.Neg
SEPT9.Neg_VIM.Pos
SEPT9.Pos_VIM.Neg
SEPT9.Pos_VIM.Pos
Component 2
Component 1
(d)
EpCAM
Relative Expression
Pseudo-time
(e)
CDH1
factor(CellType)
SEPT9.Neg_VIM.Neg
SEPT9.Neg_VIM.Pos
SEPT9.Pos_VIM.Neg
SEPT9.Pos_VIM.Pos
Component 2
Component 1
(f)
CDH1
Relative Expression
Pseudo-time

Figure S6

(a)

https://hsce.shinyapps.io/icca/

(b)

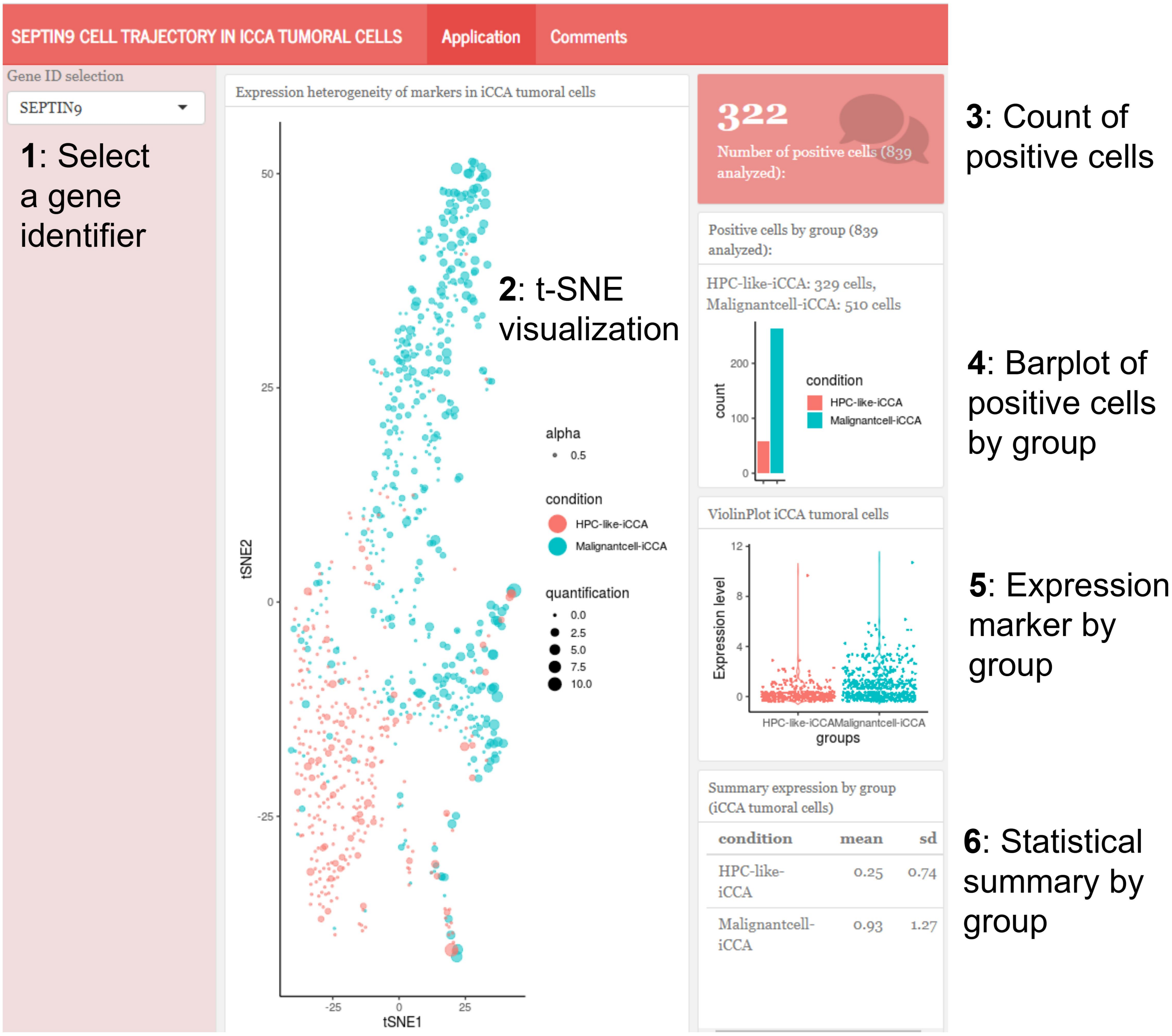

Figure S7

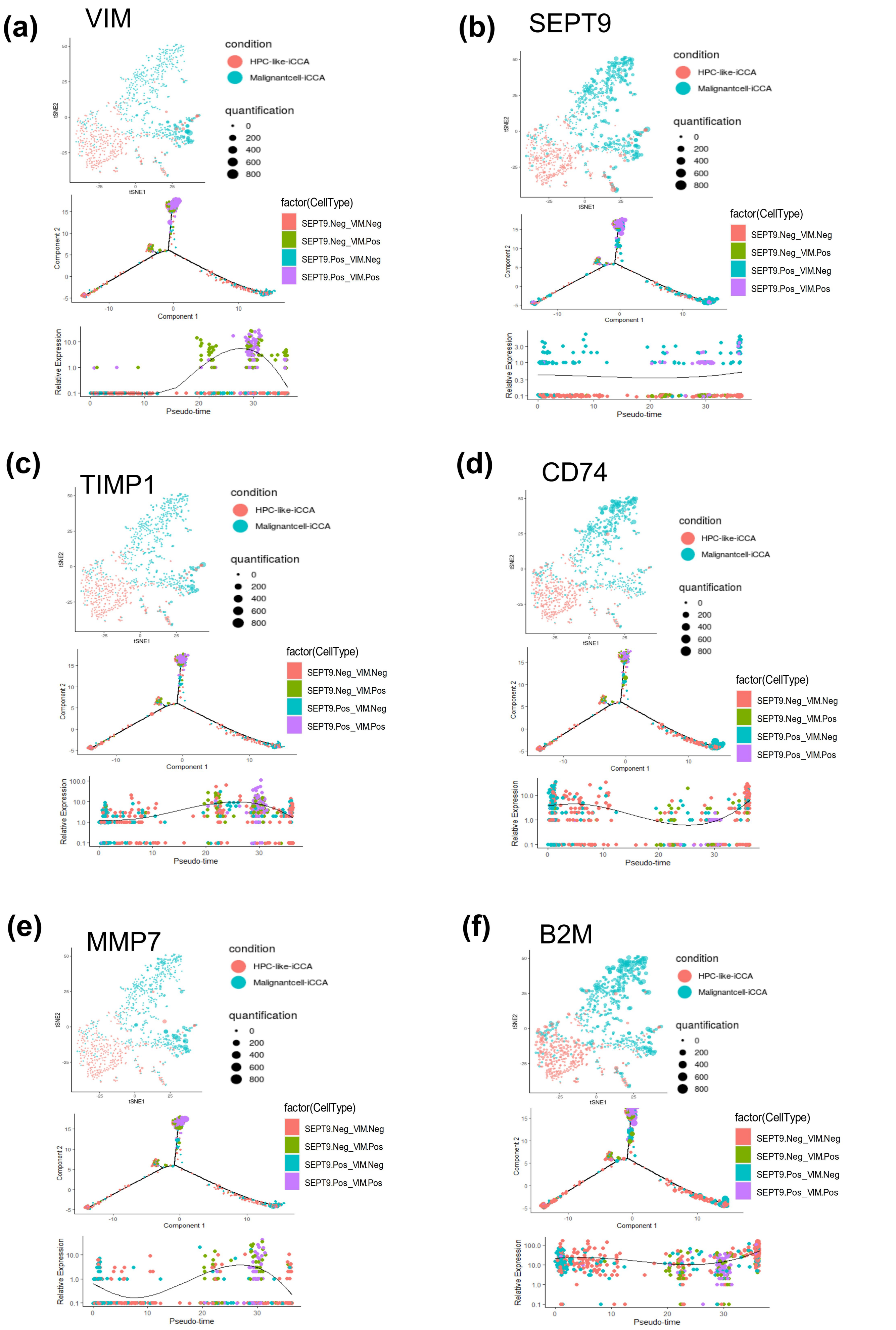
(a)
VIM
(b)
SEPT9
(c)
TIMP1
(d)
CD74
(e)
MMP7
(f)
B2M
condition
HPC-like-iCCA
Malignantcell-iCCA
quantification
0
200
400
600
800
factor(CellType)
SEPT9.Neg_VIM.Neg
SEPT9.Neg_VIM.Pos
SEPT9.Pos_VIM.Neg
SEPT9.Pos_VIM.Pos
tSNE1
tSNE2
Component 1
Component 2
Relative Expression
Pseudo-time

Figure S8

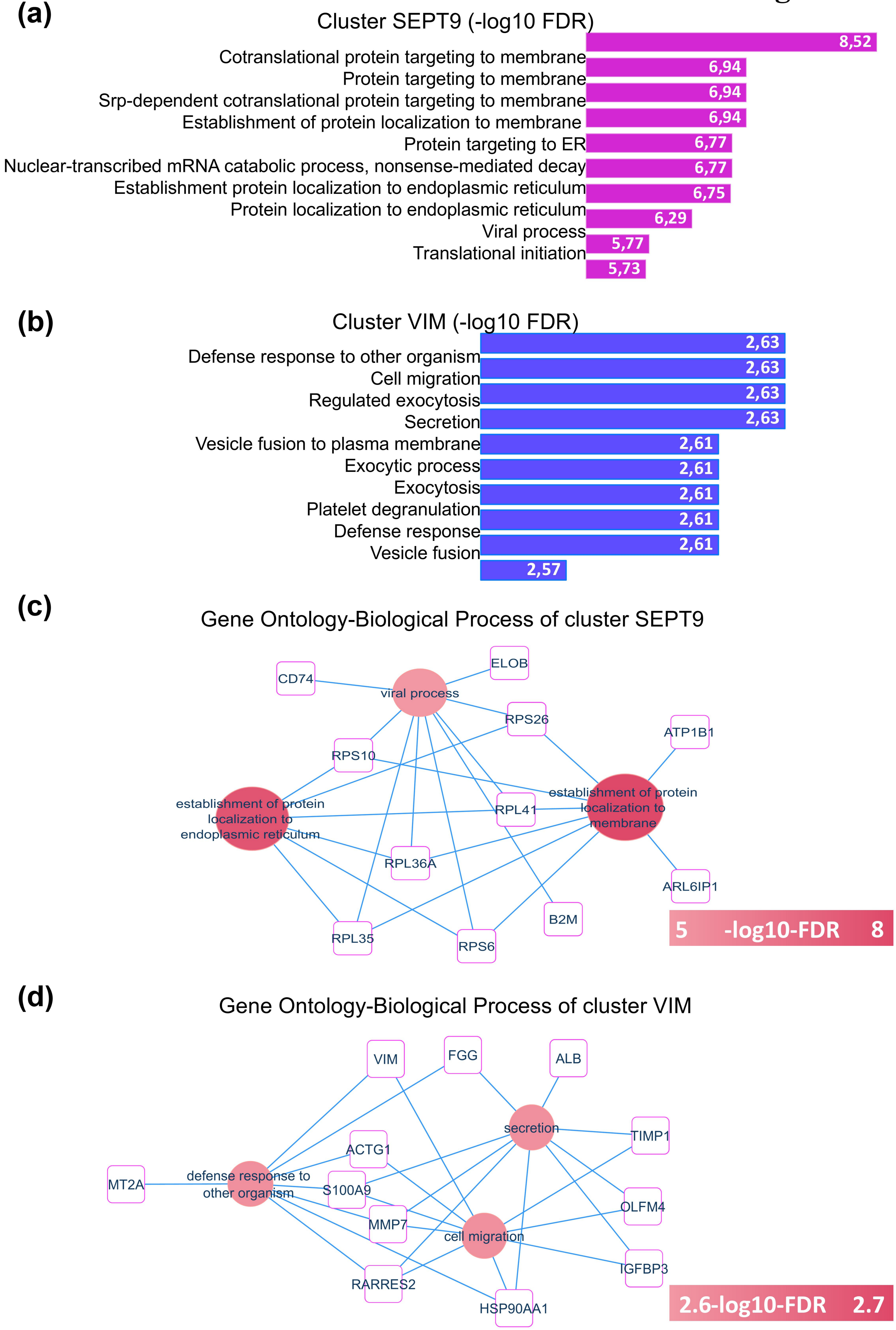
(a)
Cluster SEPT9 (-log10 FDR)
Cotranslational protein targeting to membrane
Protein targeting to membrane
Srp-dependent cotranslational protein targeting to membrane
Establishment of protein localization to membrane
Protein targeting to ER
Nuclear-transcribed mRNA catabolic process, nonsense-mediated decay
Establishment protein localization to endoplasmic reticulum
Protein localization to endoplasmic reticulum
Viral process
Translational initiation
8,52
6,94
6,94
6,94
6,77
6,77
6,75
6,29
5,77
5,73
(b)
Cluster VIM (-log10 FDR)
Defense response to other organism
Cell migration
Regulated exocytosis
Secretion
Vesicle fusion to plasma membrane
Exocytic process
Exocytosis
Platelet degranulation
Defense response
Vesicle fusion
2,63
2,63
2,63
2,63
2,61
2,61
2,61
2,61
2,61
2,57
(c)
Gene Ontology-Biological Process of cluster SEPT9
CD74
ELOB
viral process
RPS26
ATP1B1
RPS10
establishment of protein localization to endoplasmic reticulum
RPL41
establishment of protein localization to membrane
RPL36A
ARL6IP1
B2M
RPL35
RPS6
5 -log10-FDR 8
(d)
Gene Ontology-Biological Process of cluster VIM
VIM
FGG
ALB
secretion
TIMP1
ACTG1
MT2A
defense response to other organism
S100A9
MMP7
cell migration
OLFM4
IGFBP3
RARRES2
HSP90AA1
2.6-log10-FDR 2.7

Figure S9

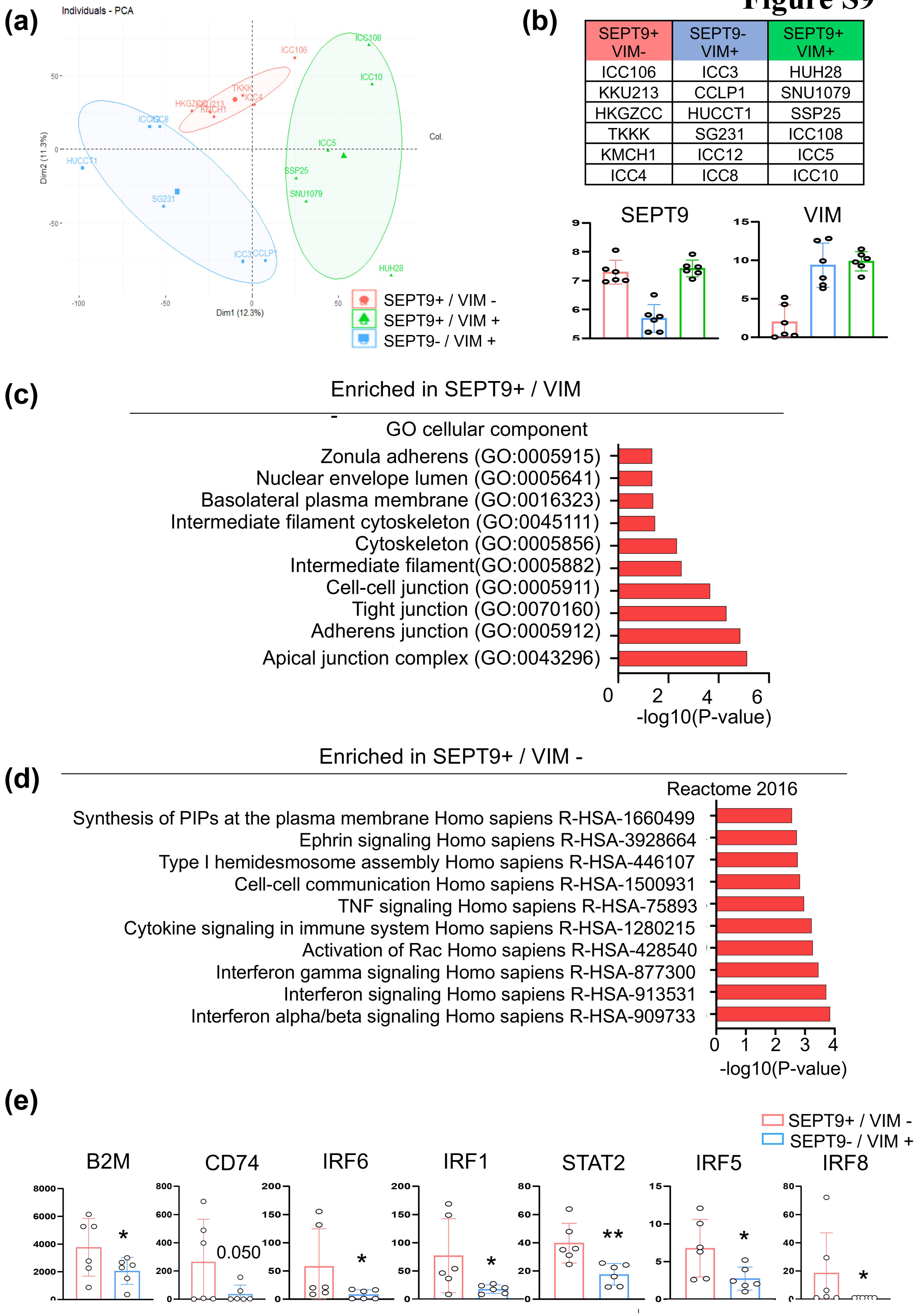
(a)
Individuals - PCA
ICC106
ICC108
ICC10
TKKK
ICC4
HKGZCC
KKU213
KMCH1
ICC12
ICC8
ICC5
HUCCT1
SSP25
SNU1079
SG231
ICC3
CCLP1
HUH28
Dim2 (11.3%)
Dim1 (12.3%)
Col.
SEPT9+ / VIM -
SEPT9+ / VIM +
SEPT9- / VIM +
(b)
SEPT9+ VIM-
SEPT9- VIM+
SEPT9+ VIM+
ICC106
ICC3
HUH28
KKU213
CCLP1
SNU1079
HKGZCC
HUCCT1
SSP25
TKKK
SG231
ICC108
KMCH1
ICC12
ICC5
ICC4
ICC8
ICC10
SEPT9
VIM
(c)
Enriched in SEPT9+ / VIM -
GO cellular component
Zonula adherens (GO:0005915)
Nuclear envelope lumen (GO:0005641)
Basolateral plasma membrane (GO:0016323)
Intermediate filament cytoskeleton (GO:0045111)
Cytoskeleton (GO:0005856)
Intermediate filament(GO:0005882)
Cell-cell junction (GO:0005911)
Tight junction (GO:0070160)
Adherens junction (GO:0005912)
Apical junction complex (GO:0043296)
-log10(P-value)
(d)
Enriched in SEPT9+ / VIM -
Reactome 2016
Synthesis of PIPs at the plasma membrane Homo sapiens R-HSA-1660499
Ephrin signaling Homo sapiens R-HSA-3928664
Type I hemidesmosome assembly Homo sapiens R-HSA-446107
Cell-cell communication Homo sapiens R-HSA-1500931
TNF signaling Homo sapiens R-HSA-75893
Cytokine signaling in immune system Homo sapiens R-HSA-1280215
Activation of Rac Homo sapiens R-HSA-428540
Interferon gamma signaling Homo sapiens R-HSA-877300
Interferon signaling Homo sapiens R-HSA-913531
Interferon alpha/beta signaling Homo sapiens R-HSA-909733
-log10(P-value)
(e)
SEPT9+ / VIM -
SEPT9- / VIM +
B2M
CD74
IRF6
IRF1
STAT2
IRF5
IRF8
0.050

Figure S10

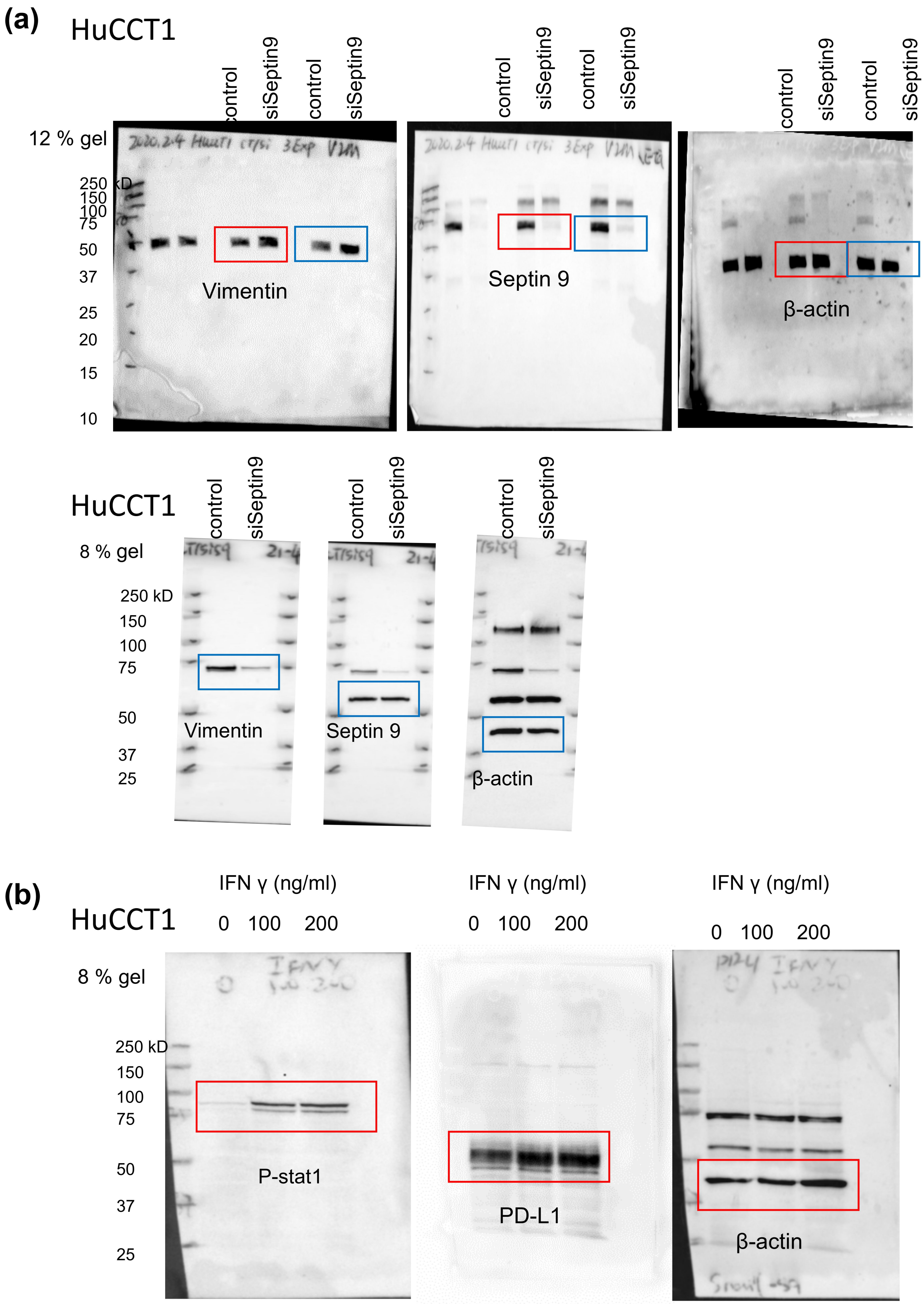
(a)
HuCCT1
control
siSeptin9
control
siSeptin9
12 % gel
250 kD
150
100
75
50
37
25
20
15
10
Vimentin
Septin 9
β-actin
HuCCT1
control
siSeptin9
8 % gel
250 kD
150
100
75
50
37
25
Vimentin
Septin 9
β-actin
(b)
HuCCT1
IFN γ (ng/ml)
0 100 200
8 % gel
250 kD
150
100
75
50
37
25
P-stat1
PD-L1
β-actin
